# Functional Analysis of Four Terpene Synthases in Rose-Scented Pelargonium Cultivars (*Pelargonium* × *hybridum*) and Evolution of Scent in the *Pelargonium* Genus

**DOI:** 10.3389/fpls.2018.01435

**Published:** 2018-11-02

**Authors:** Bernard Blerot, Laure Martinelli, Cécile Prunier, Denis Saint-Marcoux, Sylvain Legrand, Aurélie Bony, Loïc Sarrabère, Florence Gros, Nicolas Boyer, Jean-Claude Caissard, Sylvie Baudino, Frédéric Jullien

**Affiliations:** ^1^Université de Lyon, UJM-Saint-Etienne, CNRS, Laboratoire BVpam - FRE 3727, Saint-Étienne, France; ^2^IFF-LMR Naturals, Grasse, France; ^3^Université de Lille, CNRS, UMR 8198, Lille, France

**Keywords:** pelargonium, terpene synthase, essential oil, geraniol synthase, transcriptome

## Abstract

*Pelargonium* genus contains about 280 species among which at least 30 species are odorant. Aromas produced by scented species are remarkably diverse such as rose, mint, lemon, nutmeg, ginger and many others scents. Amongst odorant species, rose-scented pelargoniums, also named pelargonium rosat, are the most famous hybrids for their production of essential oil (EO), widely used by perfume and cosmetic industries. Although EO composition has been extensively studied, the underlying biosynthetic pathways and their regulation, most notably of terpenes, are largely unknown. To gain a better understanding of the terpene metabolic pathways in pelargonium rosat, we generated a transcriptome dataset of pelargonium leaf and used a candidate gene approach to functionally characterise four terpene synthases (TPSs), including a geraniol synthase, a key enzyme responsible for the biosynthesis of the main rose-scented terpenes. We also report for the first time the characterisation of a novel sesquiterpene synthase catalysing the biosynthesis of 10-epi-γ-eudesmol. We found a strong correlation between expression of the four genes encoding the respective TPSs and accumulation of the corresponding products in several pelargonium cultivars and species. Finally, using publically available RNA-Seq data and *de novo* transcriptome assemblies, we inferred a maximum likelihood phylogeny from 270 pelargonium TPSs, including the four newly discovered enzymes, providing clues about TPS evolution in the *Pelargonium* genus. Notably, we show that, by contrast to other TPSs, geraniol synthases from the TPS-g subfamily conserved their molecular function throughout evolution.

## Introduction

The *Pelargonium* genus belongs to the *Geraniaceae* family and contains about 280 species exhibiting a wild range of variation in leaf and floral morphology, as well as body plan organisation (Bakker et al., 2004; Jones et al., 2009; Roeschenbleck et al., 2014; Blerot et al., 2015). Phylogenetic analyses based on nuclear, plastidial and mitochondrial DNA led to a structuration of the *Pelargonium* genus into five main clades comprising 16 sections (Bakker et al., 2005). Although most of the odorant pelargoniums belong to clade A1 (section *Pelargonium*), some species of clade B (sections *Reniformia* and *Peristera*), clade C1 (section *Jenkinsonia*) and clade C2 (sections *Ciconium* and *Subsucculentia*) are scented (Blerot et al., 2015).

Most *Pelargonium* species are indigenous of South Africa and nearby countries. Aromas of odorant species are remarkably diverse such as rose, mint, lemon, nutmeg, ginger and many other scents, underlying a richness of scented compounds produced in *Pelargonium* (Demarne and Van der Walt, 1992; Lis-Balchin, 2003; Lalli et al., 2006). As such, several species were introduced and used in hybrids creation during the 17th century in Europe, most notably to obtain cultivars with refined fragrance notes. Among the scented *Pelargonium* hybrids, the rose scented *P.* × *hybridum* cultivars, also named pelargonium rosat, are the most emblematic cultivars and are often used to replace the expensive *Rosa damascena* essential oil (EO). These hybrids descend from several crossings between *P. graveolens* or *P. radens* in one hand and *P. capitatum* in the other hand. Cultivars used in EO production are somatically multiplied from cuttings in their production area, but the knowledge of their exact botanical origin has been lost over time (Demarne, 2003). Because of the economic importance of scented pelargoniums, agronomical research was undertaken to improve EO production and to investigate pharmaceutical and antimicrobial potentialities (Saraswathi et al., 2011; Boukhatem et al., 2013). In addition, characterisation of flavones and antioxidants in the hydrolate phase obtained during EO extraction opened new areas for valorisation of by-products (Rao et al., 2002; Sangwan and Singh, 2015).

Composition of EOs extracted from the main *P.* × *hybridum* cultivars (cv. rosat ‘Bourbon,’ cv. rosat ‘China,’ cv. rosat ‘Egypt’ and cv. rosat ‘Grasse’) has been extensively studied (Gauvin et al., 2004; Juliani et al., 2006; Blerot, 2016). Geraniol, citronellol, and derivatives, all acyclic monoterpenes, form the main part of EOs and give their stereotypical rose scent to these cultivars. In a lesser extent, limonene, a cyclic monoterpene, and its derivatives with a mint scent, as well as sesquiterpenes like 6,9-guaiadiene or 10-epi-γ-eudesmol with a rose scent and a wood scent, respectively, contribute to EOs fragrances. For a detailed composition of pelargonium rosat EOs, see Blerot (2016). Balance between these main terpenes is of crucial importance for EO quality and fragrance, with variable rosy, minty and woody top notes. As such, use and commercial value of pelargonium rosat EO depend on its composition and fragrance characteristics. For example, EO obtained from pelargonium rosat ‘Bourbon’ is much appreciated for its pure rosy fragrance.

**FIGURE 1:**
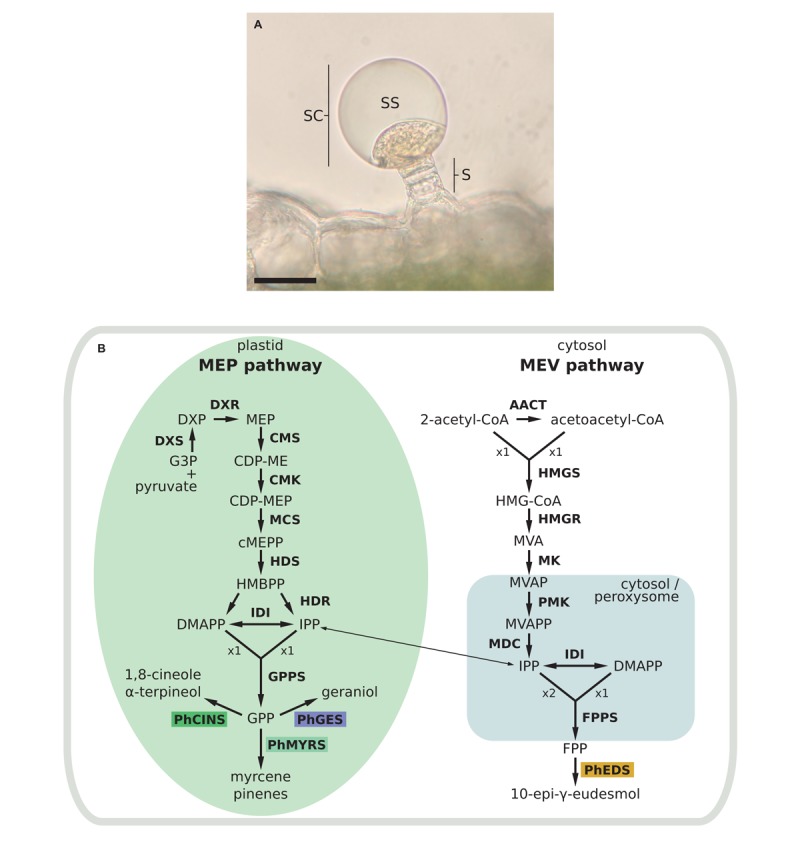
Glandular trichome and mono and sesquiterpenes biosynthetic pathways in *Pelargonium*. **(A)** Capitate secretory gland with a stack (s) constituted of several cells and surrounded by a head secretory cell (sc). Terpenes accumulate in the subcuticular space (ss). Bar scale = 10 μm. **(B)** Biosynthetic pathways of mono and sesquiterpenes. Enzymatic steps in the yellow box correspond to enzymes functionally characterised in this study. Enzymatic steps upstream of terpene biosynthesis are in bold letters. AACT, acetoacetyl-coenzyme A thiolyase; CDP-ME, 4-(cytidine 5′-diphospho)-2-*C*-methyl-D-erythritol; CDP-MEP, CDP-ME-2-phosphate; cMEPP, 2-*C*-methyl-D-erythritol 2,4-cyclodiphosphate; PhCINS, cineole synthase; CMK, 4-(cytidine 5′-diphospho) 2-*C*-methyl-D-erythritol kinase; CMS, 2-*C*-methyl-D-erythritol 4-phosphate transferase; DMAPP, dimethylallyl diphosphate; DXP, 1-deoxy-D-xylulose 5-phosphate; DXR, DXP reductoisomerase; DXS, DXP synthase; PhEDS, eudesmol synthase, FPP, farnesyl diphosphate; FPPS, FPP synthase; PhGES, geraniol synthase; G3P, glyceraldehyde 3-phosphate; GPP, geranyl diphosphate; GPPS, GPP synthase; HDS, 1-hydroxy-2-methyl-2(*E*)-butenyl 4-diphosphate synthase; HDR, 1-hydroxy-2-methyl-2(*E*)-butenyl 4-diphosphate reductase; HMBPP, 1-hydroxy-2-methyl-2(*E*)-butenyl 4-diphosphate; HMGCoA, 3-hydroxy-3-methylglutaryl-CoA; HMGR, hydroxymethylglutaryl-CoA reductase; HMGS, hydroxymethylglutaryl-CoA synthase; IDI, isopentenyl diphosphate isomerase; IPP, isopentenyl diphosphate; MDC, mevalonate pyrophosphate decarboxylase; MCS, 2-*C*-methyl-D-erythritol 2,4-cyclodiphosphate synthase; MEP, 2-*C*-methyl-D-erythritol 4 phosphate; MK, mevalonate kinase; MVA, mevalonate; MVAP, MVA-5-phosphate; MVAPP, MVA-5-diphosphate; PhMYRS, myrcene synthase; PMK, 5-phosphomevalonate kinase.

In pelargonium, biosynthesis of terpenes takes place in leaves in specialised structures known as glandular trichomes, or oil glands (Figure 1A), that are composed of a secretory cell producing EO in a subcuticular storage cavity (Boukhris et al., 2013). Within the secretory cell, prenyl phosphate like isopentenyl diphosphate (IPP) and its allylic isomer, dimethylallyl diphosphate (DMAPP) are biosynthesised through both the cytosolic mevalonate (MVA) and the plastidial methyl erythritol phosphate (MEP) pathways (Figure 1B). It is generally accepted that IPP/DMAPP provided by MEP and MVA pathways are used, respectively, for the biosynthesis of monoterpenes and sesquiterpenes, but several works have pointed out cross-talks between these two metabolic routes (Opitz et al., 2014; Mendoza-Poudereux et al., 2015). IPP/DMAPP are substrates of prenyltransferases, enzymes involved in hydrocarbon chain elongation of prenylated compounds. The head-to-tail condensation of one DMAPP molecule with one IPP molecule produces geranyl diphosphate (GPP). Adding another IPP unit to GPP produces farnesyl diphosphate (FPP). Longer prenyl diphosphates can be synthesised but are not used to produce volatile compounds.

Prenyl diphosphates are substrates of terpene synthases (TPSs), a class of enzymes found in bacteria, fungi, plants, as well as some organisms from *Excavata* and *Amoebozoa* eukaryotic supergroups (Bohlmann et al., 1998; Wawrzyn et al., 2012; Yamada et al., 2015; Chen et al., 2016). In plants, TPSs likely evolved from the duplication of a gene encoding for an enzyme catalysing the formation of *ent*-kaurene, a precursor in the gibberellin pathway (Hayashi et al., 2006). TPSs including both monoterpene synthases (mTPSs), sesquiterpene synthases (sTPSs), and diterpene synthases (diTPSs) can be divided in seven subfamilies, of which five are represented in angiosperms: TPS-a contains sTPSs, TPS-b mTPSs, TPS-c diTPSs, TPS-e/f and TPS-g mTPSs, sTPSs and diTPSs (Chen et al., 2011). Conversion of GPP and FPP in terpenes is ensured by mTPSs and sTPSs, respectively, although some enzymes are able to accept both substrates when the reaction occurs *in vitro* (Nagegowda et al., 2008). The catalytic step mediated by TPSs consists in the dephosphorylation of a prenyl diphosphate molecule, thus forming an instable intermediary carbocation, followed by molecular rearrangements of the carbocation until loss of a proton or addition of water ends the reaction. A magnesium ion is needed for the dephosphorylation step and two conserved motifs, DDxxD and NSE/DTE are known to help for Mg^2+^ ion stabilisation (Degenhardt et al., 2009). A third conserved motif, RR(x_8_)W, facilitates the cyclisation of carbocations (Williams et al., 1998). Many TPSs are promiscuous enzymes forming a large number of terpene products owing to their catalytic mechanism (Tholl et al., 2005).

Primary products of TPSs can undergo several rounds of subsequent modifications, thus producing the huge terpene diversity observed in plants. For example, in pelargonium, geraniol can be transformed by a multistep enzymatic pathway in a series of odorant compounds like geranial, geranyl acetate, and geranyl formate in one hand, and citronellol and derivatives in another hand. Compared to the full range of terpene secondary modifications known so far, only a few genes encoding enzymes involved in these reactions have been characterised. For example, oxidation of geraniol in geranial is mediated by an alcohol dehydrogenase (ADH) in basil (Iijima et al., 2006), ginger (Iijima et al., 2014), perilla (Sato-Masumoto and Ito, 2014), the orchid *Caladenia* (Xu et al., 2017) and *Polygonum* (Hassan et al., 2012). Gene encoding for the enzyme mediating acetylation of geraniol and citronellol has only been characterised once in rose (Shalit et al., 2003). Complicating the identification of enzymes involved in terpene secondary modifications, the same molecule can be obtained by different molecular routes. For example, citronellol biosynthesis occurs either by a direct reduction of geraniol or through a multistep pathway involving both ADH and reductase enzymes (Iijima et al., 2014; Xu et al., 2017).

Balance between primary terpenes and their secondary derivatives largely influences the fragrance produced by aromatic plants. Regulation of TPS and other enzymes involved in terpene biosynthesis is both controlled transcriptionally (Nagegowda, 2010) and by IPP/DMAPP supply (Muñoz-Bertomeu et al., 2006). In pelargonium, a gene encoding for 1-deoxy-D-xylulose-5-phosphate synthase (DXS), the first step providing IPP/DMAPP by the MEP pathway, has been characterised. Overexpression of the *DXS* gene led to a slight increase in EO content (Jadaun et al., 2017).

In aromatic non-model plants, the first EST library was derived from isolated secretory trichomes of peppermint (Lange et al., 2000). This strategic resource allowed the characterisation of reductases (Davis et al., 2005; Ringer et al., 2003), dehydrogenases (Ringer et al., 2005) and cytochrome P450 monooxygenases (Bertea et al., 2001), all involved in the menthol biosynthetic pathway. Several other libraries were obtained from purified glands of basil (Gang et al., 2001), oregano (Crocoll et al., 2010) and lavender (Lane et al., 2010) resulting in gene characterisation in both isoprenoid and phenylpropanoid biosynthetic pathways. Transcriptome investigation using high throughput sequencing technologies (RNA-Seq) has opened an entire new era in biology, allowing for large scale studies of gene expression, even in non-model organisms for which no genome has been sequenced. In the specialised metabolism field of research, RNA-Seq not only allowed to decipher entire metabolic pathways (Dugé de Bernonville et al., 2015), but also their regulators like transcription factors (Wang et al., 2016). However, pelargonium sequence data are yet scarce. Illumina reads for two dozen accessions of *Geraniales*, of which half are *Pelargonium* species, are publically available. Although this dataset encompasses several odorant pelargoniums, TPSs were not investigated. Recently, the first transcriptome analysis of a pelargonium rosat (cv. ‘Bourbon’) was reported (Narnoliya et al., 2017) and 13 mTPSs, 5 sTPSs, and 10 dTPSs sequences were identified solely by sequence homology.

In this study, we report the identification and functional characterisation of a geraniol synthase (PhGES) and an eudesmol synthase (PhEDS), two major TPSs controlling EO composition in pelargonium rosat cultivars, as well as a cineole and a myrcene synthase (PhCINS and PhMYRS, respectively) using a 454 transcriptome of *P*. × *hybridum* cv. rosat ‘Grasse.’ We show a strong correlation between expression of the genes encoding these enzymes and presence of their respective products in several pelargonium cultivars and species. Finally, using publically available RNA-Seq data and *de novo* transcriptome assemblies, we place the four enzymes in an evolutionary perspective of the TPSs in the *Pelargonium* genus.

## Materials and Methods

### Plant Material

*P.* × *hybridum* cv. rosat ‘Grasse,’ *P.* × *hybridum* cv. rosat ‘Egypt,’ *P.* × *hybridum* cv. rosat ‘Bourbon,’ *P.* × *hybridum* cv. rosat ‘China’ and *P.* cv. ‘Toussaint,’ were collected in their country of origin by IFF/LMR Naturals. *P.* cv. ‘clorinda,’ *P. tomentosum*, *P. radens*, *P. graveolens*, *P. capitatum*, *P. quercifolium*, were supplied by ‘Le Conservatoire National du *Pelargonium*, Bourges’ and ‘Le Jardin Botanique de la tête d’Or, Lyon.’ All plants were cultivated in a green house in the laboratory BVpam in Saint-Étienne under a photoperiod of 16 h of light versus 8 h of dark. Temperature was 22.5°C in the light and 18°C in the dark. Hygrometry was 50% and stable all over the day. *P. citronellum*, *P. australe*, *P. cotyledonis*, *P. echinatum*, *P. exstipulatum*, *P. fulgidum*, *P. hortorum*, *P. tetragonum*, and *P. transvaalense* leaves were collected from ‘Le jardin botanique de la tête d’Or, Lyon.’

### RNA Extraction

Total RNA extraction was performed using a modified version from Chang et al. (1993) and Cock et al. (1997). Therefore, 1 g of young leaves were ground to powder in liquid nitrogen before addition of 5 mL of extraction buffer [2% (w/v) PVP; 2% (w/v) CTAB; 100 mM Tris-HCl pH 8; 25 mM EDTA pH 8; 2 M NaCl] and 100 μL of β-mercaptoethanol. Homogenised sample was incubated 15 min at 65°C followed by 1 min at room temperature and then extracted twice with an equal volume of chloroform:isoamylic alcohol (24:1). Supernatant (aqueous phase) was collected by centrifugation at 9,000 × *g* for 20 min at 4°C. RNA was precipitated by the addition of lithium chloride (2 M) and incubated for 24 h at 4°C. After centrifugation at 14,000 rpm for 1 h at 4°C, pellet was washed twice with ice cold ethanol 70% and air dried. Pellet was dissolved with 25 μL of water and 500 μL of SSTE buffer [1 M NaCl; 0.5% (w/v) SDS; 10 mM Tris-HCl pH 8; 1 mM EDTA pH 8]. Aqueous phase was collected by centrifugation at 14,000 rpm for 10 min at 4°C and RNA was precipitated by the addition of 1 mL of ethanol 100% and 150 μL of sodium acetate 3 M (pH 5.7). RNA pellet was harvested after overnight incubation at −20°C and centrifugation at 14,000 rpm for 30 min at 4°C. Pellet was then washed twice with ice cold ethanol 70%, air dried and solubilised in pure water.

### Genome Walking

One region of the *PhGES* gene, the promoter and the ATG start site, were amplified using the GenomeWalker^TM^ kit (Clontech, cat. # 638904) according to manufacturer’s instructions. DNA samples were extracted from leaves using NucleoSpin^®^ Plant II Genomic DNA (Macherey-Nagel, cat. # 740770) according to manufacturer’s instructions. As recommended by the GenomeWalker^TM^ kit, PCR and nested-PCR were performed using Advantage 2 PCR kit (Clontech, cat. # 639207). Primers were designed following the guidelines from the kit and are listed in Supplementary Table 1.

### 454 Pyrosequencing Library and Reads Analysis

Total RNA from *P.* × *hybridum* rosat ‘Grasse’ was extracted from young leaves using the Tri reagent kit according to the manufacturer’s instructions and 75 μg was sent to Eurofins MWG GmbH. A normalised random-primed cDNA library was prepared from the RNA, an emulsion-based PCR was performed and one segment of a sequencing plate was sequenced on a GS FLX+ (454/Roche) to yield more than 698,000 reads delivered as assembled reads in FASTA format with quality scoring files of all clusters. Reads were deposited in the SRA under the number SRP144736. Cleaned reads were checked using fastQC^1^ and assembled using CAP3 (Huang and Madan, 1999) with a minimum similarity threshold of 90% and a minimum overlap of 40 bases. Assembled sequences were annotated using Blastx searches performed against the *Arabidopsis* translated coding sequences [The Arabidopsis Information Resource (TAIR), V10^2^]. Several Perl scripts were designed for data processing and analysis in assembling, annotation and functional classification of the sequences.

### *De novo* Transcriptome Assembly From Publicly Available Illumina Reads

Illumina paired-end reads were downloaded from the SRA database^3^. *P.* × *hybridum* cv. rosat ‘Bourbon’ (SRR3747647) reads were obtained from the project SRP078041 (Narnoliya et al., 2017), *P. exstipulatum* (SRR1856381), *P. echinatum* (SRR1856382), *P. myrrhifolium* (SRR1856383), *P. citronellum* (SRR1862850), *P. cotyledonis* (SRR1863089), *P. incrassatum* (SRR1864553), *P. fulgidum* (SRR1864810), *P. dichondrifolium* (SRR1864811), *P. nanum* (SRR1865090), *P. australe* (SRR1865110), *P. transvaalense* (SRR1865111), *P. tetragonum* (SRR1865385) reads were obtained from the project SRP055845, *P.* × *hortorum* reads (SRR1072946 and SRR498624) were obtained from the project SRP013265. Reads were quality checked using fastQC and cleaned using Trimmomatic v0.36 (Bolger et al., 2014) and a collection of custom Perl scripts. Clean reads were assembled using a pipeline based on SGA assembler (Simpson and Durbin, 2011) as described in Bonnot et al. (2017). Coding sequences were predicted from transcripts using a pipeline based on interproscan 5.24–63.0, Blast v2.6.0 and TransDecoder v3.0.1^4^ softwares.

### Phylogenetic Analyses

Terpene synthases sequences were fetched in pelargonium assembled transcriptomes using hmmer v3.1b2 (Eddy, 2011) and the HMM profile Terpene_synth_C (PF03936) from PFAM database corresponding to the α domain found in the C-terminal part of plant enzymes. Short peptides and sequences devoid of the DDxxD conserved motif typical of class I TPSs were removed. Sequences were aligned with clustalω v1.2.4 (Sievers et al., 2011) using the Terpene_synth_C hmm profile as a guide and 10 iterations. Some sequences obviously belonging to the same gene were manually joined together. During exploratory work, FasttreeMP v2.1.10 (Price et al., 2010) was used to generate phylogenies. Sequences of functionally characterised enzymes from neighbour families of *Pelargonium* genus and from *Arabidopsis* were added to the tree in order to tentatively assigned functions to groups of *Pelargonium* TPSs (Supplementary Table 2); nearly identical sequences with the same biochemical function were omitted. Sequences not belonging to TPS clades -a, -b, -g or -e/f (Chen et al., 2011) were removed from the phylogeny. Final alignment was manually edited to remove non-homologous sites and obvious misalignments (Supplementary File 1). The final tree was obtained using RAxML pthreads v8.2.11 (Stamatakis, 2014), the JTT evolutionary model, four gamma categories and amino acids frequencies deduced from the alignment as determined with the help of Prottest v3.4.2 (Darriba et al., 2011). Bootstrap values were obtained during the RAxML run until convergence (300 bootstraps). Pelargonium TPSs were blasted against NCBI nr database and results were compiled from the first 50 hits to obtain a functional annotation of the sequences.

### Expression of Recombinant PhGES, PhEDS, PhCINS, and PhMYRS in *E. coli*

Full length TPSs genes amplified using primers listed in Supplementary Table 1 were inserted into the vector pENTR/D-TOPO and transferred by homologous recombination in Busso expressing vectors (Busso et al., 2005). Primers used for these steps are described in Supplementary Table 1. *E. coli* strain Rosetta (DE3) pLysS cells (Novagen, Darmstadt, Germany) were then transformed with the expression vector by heat shock. Production of the heterologous protein was performed during 14–16 h at 16°C under constant shaking at 180 rpm in terrific broth supplemented with 0.5% glycerol, 0.25 M D-sorbitol, 2.5 mM betaine after induction with 0.2 mM IPTG. The cells recovered by centrifugation were disrupted by sonication in native binding buffer (50 mM NaH_2_PO_4_, 0.5 M NaCl, 20 mM imidazole, 5% glycerol, 5 mM DTT, pH 8) supplemented with 0.5 mg.mL^−1^ lysozyme. After clarification of the lysate by centrifugation, the recombinant protein was purified by binding to the Talon^®^ metal affinity resin (Clontech, cat. # 635515) according to the manufacturer’s instruction. The resin-bound protein was incubated overnight at 4°C in 200 μL of native binding buffer supplemented with 10 units of thrombin. The next day the TPS was recovered from the mixture by filtration. Protein concentration was measured using the Bio-Rad reagent (cat. # 500-0006) with bovine serum albumin as standard (Bradford, 1976).

Enzymatic assays were performed at least in three replicates in a final volume of 500 μL containing 15–50 μg of purified recombinant protein, buffer (25 mM Tris-Cl, pH 7.5, 10% glycerol, 1 mM DTT, 1 mg.mL^−1^ BSA) and cofactors (10 mM MgCl_2_, 1 mM MnCl_2_). The reaction was initiated by addition of 50 μM geranyl or FPP and the mixture was overlaid with 500 μL of hexane. After 2 h incubation at 30°C, the mixture was vigorously mixed and the upper hexane phase was collected, concentrated under nitrogen stream and analysed by GC/MS. Negative controls were performed using the purified product from Rosetta (DE3) pLysS without expression vector.

### Subcellular Localisation

*In silico* predictions of proteins subcellular localisation were made with TargetP (Emanuelsson et al., 2007) and Predotar (Small et al., 2004) web services. *PhGES*, *PhCINS*, *PhMYRS* and *PhEDS* full length coding sequences were amplified using primers reported in Supplementary Table 1. Following cloning in the pENTR/D-TOPO entry vector, sequences were transferred into the expression vector pMDC83 under the control of a double 35S promoter (Curtis and Grossniklaus, 2003), to generate a fusion protein with GFP fused to the C-terminal part of the TPS protein. This construct was transformed into the *Agrobacterium tumefaciens* strain C58 (pMP90). Agrobacteria cultures expressing independently the four TPSs were co-infiltrated in *Nicotiana benthamiana* leaves according to Batoko et al. (2000) together with agrobacteria expressing the P19 viral suppressor of silencing (Voinnet et al., 2003). After 5 days, infiltrated leaf sectors were observed under a confocal microscope as reported in Magnard et al. (2015).

### RT-PCR Analysis

Quantitative PCR was performed with CFX96^TM^ real-time detection system (Bio-Rad, Hercules, CA, United States) using the SsoAdvanced^TM^ SYBR Green Supermix (Bio-Rad, cat. # 172-5270). All reactions were carried out in 20 μL using 2 μL of reverse transcribed cDNA as template and 500 nM of each of the primers according to the manufacturer’s protocol. Gene primers were designed using the Primer3 software. Two biological replicates were analysed for each accession then cDNA was amplified twice in two independent qPCR runs with each primer combination. Therefore, data yielded four replicates per original sample. The following thermal profile was used for all PCR reactions: 95°C for 30 s, followed by 40 quantification cycles [95°C for 5 s, Tm (Supplementary Table 1) for 30 s]. After 40 cycles, a melting-curve analysis (65°C to 95°C, one fluorescence read every 0.5°C) was performed to check the specificity of the amplification.

Normalised expression values (2^−ΔΔCq^ method, Livak and Schmittgen, 2001) of *PhGES* and *PhEDS* were calculated by the CFX96TM data manager (Bio-Rad, Hercules, CA, United States) using β-*ACTIN*, *TUBULIN* and *GAPDH* as reference genes (Supplementary Table 1). The stability of expression of these reference genes was evaluated using Best-Keeper (Pfaffl et al., 2004), geNorm v. 3.5 (Vandesompele et al., 2002), and NormFinder (Andersen et al., 2004). We used REST 2009 (Pfaffl et al., 2002) to compare the expression level of a gene in a ‘sample’ group using a ‘control’ group as a reference by implementing a pairwise fixed reallocation randomization test (10,000 iterations). Differences in expression between ‘sample’ and ‘control’ cDNAs were considered significant for *p*-values < 0.05.

Semi-quantitative RT-PCR was performed using primers listed in Supplementary Table 1 and samples were normalised using *ACTIN* gene. After 25 amplification cycles, amplification levels were estimated following in-gel staining and visual comparison against *ACTIN* gene amplification.

### GC-MS Analysis

Terpenes were extracted independently from fresh leaves of three plants after overnight incubation in hexane (2 mL per gram), supplemented with camphor as internal standard to allow for quantification, or by hydro-distillation. An Agilent GC 6850 gas chromatograph coupled with an Agilent 5973 ion trap mass detector was used for GC-MS analyses of enzymatic activities and transiently transformed tobacco leaf extracts. The instrument was equipped with a 30 m × 0.25 mm apolar capillary column DB5. Temperatures of injector and detector were 250°C. Helium was used as the carrier gas at a flow rate of 1.0 mL.min^−1^. A volume of 2 μL of extract was injected with a split ratio of 1:2. Oven temperature settings were: 4 min at 60°C after injection followed by a 4°C.min^−1^ temperature ramp from 60°C to 240°C. Temperature was then kept on hold at 240°C for 5 min. Molecule identification was performed using Wiley, NIST 05 and IFF-LMR mass spectra databases. GC-MS analyses in IFF analytic laboratory (Grasse) were performed on a GC-MS 6890-MS Agilent 5973. Most of the parameters were common with those applied in LBVpam except for the temperature ramp that was 60°C during 10 min, then 2°C.min^−1^ from 60°C to 300°C. Temperature was then kept on hold at 300°C for 3 min. A volume of 2 μL of extract was injected with a split ratio of 1:110.

## Results

### Functional Characterisation of a Geraniol Synthase and an Eudesmol Synthase, as Well as Two mTPSs From *P. × hybridum* Rosat ‘Grasse’

**FIGURE 2:**
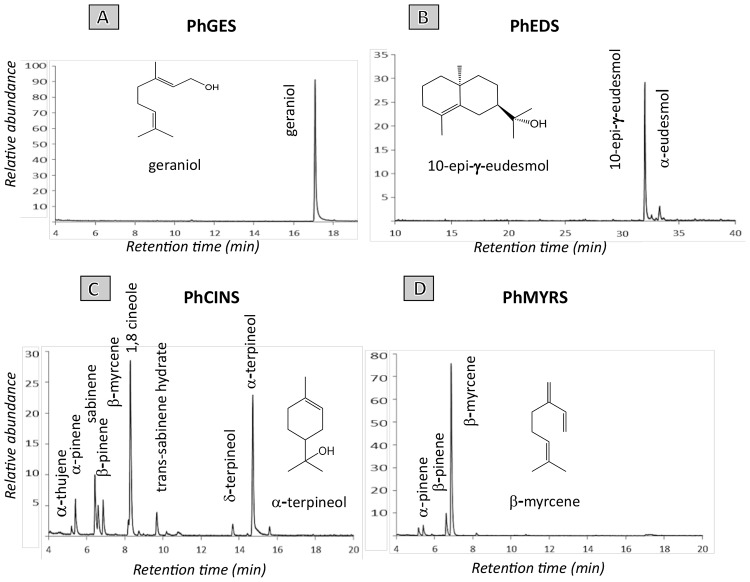
GC-MS analysis of hexane extracts obtained after incubation of PhGES **(A)**, and PhEDS **(B)**, respectively, with GPP and FPP. Enzymatic activity of both PhCINS **(C)** and PhMYRS **(D)** were characterised after incubation with GPP. Compounds identification was determined by comparison to mass spectra databases (Wiley, NIST, and IFF) and linear retention indices. A comparison of mass spectrum between main products of each enzyme and standards is shown in Supplementary Figure 1. Enzymatic assays of both PhEDS and PhGES were performed in three independent experiments.

Geraniol, limonene, eudesmol and their derivatives make up to 75% of EO composition in pelargonium rosat cultivars. As such, characterising TPSs responsible for their synthesis is an important step to better understand how these terpenes are produced in pelargonium. To this aim, a transcriptome of *P.* × *hybridum* rosat ‘Grasse’ was *de novo* assembled from 454 reads obtained from leaf RNA. Known sequences of GES, limonene synthase and EDS were used to search for homologous sequences in the transcriptome and four TPSs were identified as good candidates based on their percentage of similarity. The four sequences were cloned and recombinant proteins were produced in the *E coli* Rosetta^TM^ (DE3) pLysS strain using Busso’s expression vectors (Busso et al., 2005). Incubation of the four recombinant proteins with GPP or FPP led to the identification of three mTPSs and one sTPS. PhGES (MF503883) produced uniquely geraniol in presence of GPP as a substrate (Figure 2A). PhEDS (MF503882) produced 10-epi-γ-eudesmol and α-eudesmol as major and minor products, respectively, after incubation with FPP (Figure 2B). The two remaining enzymes were multiproduct mTPSs: PhCINS (MF503881) catalysed the *in vitro* production of more than 10 monoterpenes from GPP, with 1,8-cineole and α-terpineol as major products (Figure 2C); PhMYRS (MF503884) used GPP to produce β-myrcene as major compound and both α- and β-pinene in lower amounts (Figure 2D). Unfortunately, none of the enzymes catalysed the formation of limonene. No sesquiterpene could be detected when PhGES, PhCINS or PhMYRS were incubated with FPP; similarly, PhEDS was unable to catalyse the production of any monoterpene in presence of GPP. Retention times of all enzymatic products were similar to standards (data not shown) and a comparison of mass spectrum between enzymatic products and standards was also performed (Supplementary Figure 1).

### Sequence Analysis of PhGES, PhEDS, PhCINS, and PhMYRS

**FIGURE 3:**
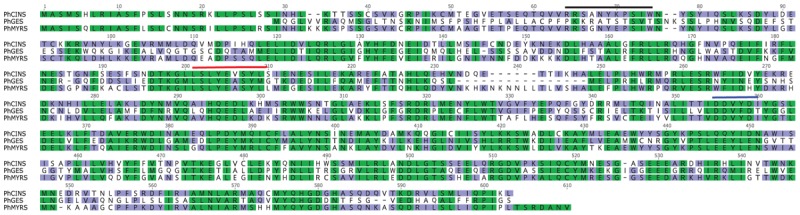
Protein sequence alignment of the three mTPSs isolated from *P.* × *hybridum* cv. rosat ‘Grasse,’ *PhCINS* (MF503881), *PhGES* (MF503883) and *PhMYRS* (MF503884). Conserved amino acid regions typical of terpene synthases are overlined: motif RR(x)_8_W in black, motif LSLYEASYL in red and motif DDxxD in blue.

**FIGURE 4:**
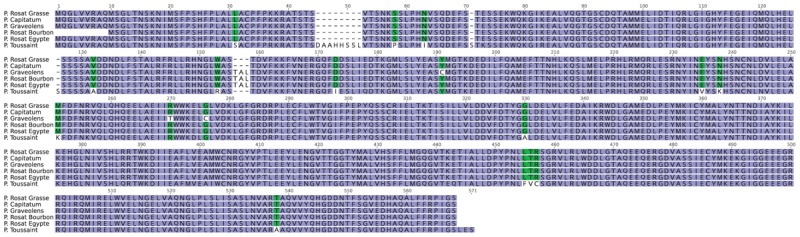
Protein sequence alignment of GESs from different pelargonium rosat cultivars, *P.* cv. ‘Toussaint’ and two pelargonium rosat parents: *P. capitatum* and *P. graveolens*.

Alignment of the protein sequences of the three mTPS showed a strong similarity between PhCINS and PhMYRS (identity: 63%; similarity: 77%) compared to PhGES (Figure 3). The three enzymes contained conserved motifs of TPSs: LSLYEASYL and DDxxD; RR(x)_8_W, was absent from the PhGES sequence, as expected for a mTPS catalysing the synthesis of acyclic products like geraniol. Because geraniol and derivatives are the main terpenes responsible for the rose scent of pelargonium rosat, orthologs of PhGES in several rosat cultivars as well as in their parents were examined (Figure 4). PhGES sequence was highly conserved between the different pelargonium rosat as compared to *P.* cv. ‘Toussaint.’ A sequence of three amino acids (TAL) was only found in ‘Bourbon’ and ‘Egypt’ cultivars but not in ‘Grasse’ cultivar. *PhGES* and *PhEDS* gene structures were investigated by amplifying genomic DNA: *PhGES* was composed of seven exons and had a gene length of 3,640 bp whereas *PhEDS* was composed of eight exons for a gene length of 2,681 bp (Supplementary Table 3). Exon size was conserved between the two genes whereas introns were longer on average in *PhEDS*. *PhGES* gene structure is in accordance with previous published data of monoterpene genomic structure (Martin et al., 2010), except for an unusual long intron of 542 bp in position three.

### *In vivo* Localisation of the Four TPSs

**FIGURE 5:**
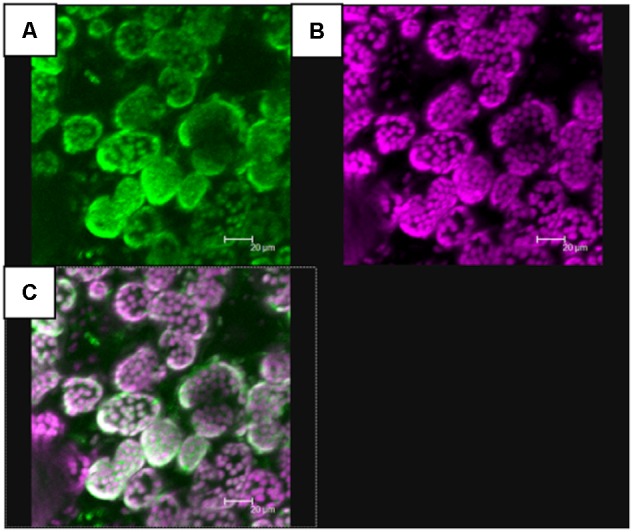
*In vivo* localisation of PhGES by transient transformation of *N. benthamiana* with *Agrobacterium tumefaciens* carrying a pMDC32 expression vector where PhGES coding sequence, was fused with the GFP coding sequence. PhGES was localised in chloroplasts. Two independent experiments were performed and chloroplastic localisation was found at least in several plants in both assays. Picture shows the green fluorescence of GFP **(A)**, auto fluorescence of chlorophyll in purple **(B)**, and their co-localisation **(C)**.

It is generally believed that the plastidial MEP pathway and the cytosolic MVA pathway provide IPP/DMAPP for the synthesis of monoterpenes and sesquiterpenes, respectively. As such, mTPSs are expected to be localised in the plastid, whereas sTPSs are expected to be cytosolic. *In silico* targeting predictions of PhGES, PhCINS, and PhMYRS indicated that the three enzymes were targeted to the plastid, excepted PhGES predicted to be localised in the mitochondria solely by Predotar software (not shown). To verify these predictions, *N. benthamiana* leaves were transiently transformed with each TPS fused with the GFP moiety at the end of their C-terminal (Curtis and Grossniklaus, 2003). Confocal microscopy showed that PhGES was localised in the plastid (Figure 5) as well as PhCINS (Supplementary Figure 2). Our attempt to localise PhMYRS was unsuccessful but its high similarity with PhCINS (Figure 3) led us to be confident with *in silico* predictions. Transformed plants only with agrobacteria expressing the P19 viral suppressor of silencing provided a negative control of GFP fluorescence. Unexpectedly, PhEDS was predicted to be localised in the plastid, but all targeting predictions overlapped with the RR(x)_8_W conserved motif involved in the biosynthesis of cyclic sesquiterpenes like eudesmol, thus indicating a misprediction of PhEDS subcellular localisation. Unfortunately, our attempts to localise PhEDS *in vivo* were unsuccessful.

### Correlation of Expression of the Four TPSs With Terpene Content in Different *Pelargonium* Species

To validate PhGES, PhEDS, PhCINS, and PhMYRS functions *in vivo*, terpene content and expression of the four enzymes were investigated jointly in several *Pelargonium* species. Several *P.* × *hybridum* cultivars, their putative parents, *P. capitatum*, *P. graveolens* and *P. radens*, as well as some botanical species were analysed (Table 1). Terpene amount ranged from 0.5 mg.g^−1^ FW up to 2.7 mg.g^−1^ FW depending on the species. *P. graveolens* produced geraniol and both *P. graveolens* and *P. capitatum* produced citronellol and derivatives, giving a rose and lemon scent to these species. *P. radens* contained almost exclusively menthone and its derivatives relative to the other terpenes detected. Pelargonium rosat cultivars synthesised both rose and mint scented terpenes with different balances between geraniol and citronellol. Finally, *P*. cv. ‘Clorinda,’ *P. quercifolium* and *P. tomentosum* exhibited a different pattern of terpenes with a higher amount of non-oxygenated monoterpenes like myrcene, phellandrenes and ocimenes.

**Table 1:** Percentages of terpene compounds and total amount of terpenes (mg.g^−1^ FW) in several pelargonium species and cultivars.

	*P. cap*	*P. grav*	*P. radens*	*P. Bour*	*P. Grasse*	*P. Egypt*	*P. Clor*	*P. quer*	*P. tom*
Pinenes	0.61 ± 0.07	0.4 ± 0.02	0.00 ± 0.00	0.56 ± 0.07	0.48 ± 0.01	0.49 ± 0.03	4.57 ± 0.07	0.36 ± 0.03	0.52 ± 0.01
Myrcene	0.00 ± 0.00	0.00 ± 0.00	1.18 ± 0.16	0.21 ± 0.11	0.00 ± 0.00	0.11 ± 0.06	15.70 ± 0.56	1.01 ± 0.26	3.54 ± 0.14
*p*-Cymene	0.45 ± 0.05	0.00 ± 0.00	0.42 ± 0.04	0.00 ± 0.00	0.00 ± 0.00	0.00 ± 0.00	2.38 ± 0.98	17.31 ± 4.45	4.16 ± 1.08
Phellandrene	0.46 ± 0.07	0.00 ± 0.00	0.85 ± 0.09	0.16 ± 0.09	0.06 ± 0.03	0.03 ± 0.03	22.64 ± 1.22	12.2 ± 2.78	19.27 ± 0.23
Ocimenes	0.57 ± 0.18	0.00 ± 0.00	1.59 ± 0.32	0.21 ± 0.1	0.00 ± 0.00	0.00 ± 0.00	0.00 ± 0.00	17.54 ± 1.17	0.00 ± 0.00
Linalol	0.00 ± 0.00	0.00 ± 0.00	0.5 ± 0.01	0.07 ± 0.07	0.21 ± 0.12	0.23 ± 0.06	6.30 ± 0.41	0.00 ± 0.00	0.00 ± 0.00
Menthone	0.00 ± 0.00	5.85 ± 0.29	88.87 ± 0.77	11.41 ± 0.72	9.44 ± 0.75	8.07 ± 0.07	2.21 ± 0.04	0.00 ± 0.00	57.2 ± 0.97
Citronellol	26.68 ± 3.88	40.85 ± 0.85	0.00 ± 0.00	28.64 ± 2.44	32.12 ± 2.79	44.63 ± 0.53	0.00 ± 0.00	0.00 ± 0.00	0.57 ± 0.14
Geraniol	0.21 ± 0.12	17.89 ± 0.24	0.00 ± 0.00	38.82 ± 3.45	40.12 ± 3.88	25.56 ± 0.61	0.00 ± 0.00	0.00 ± 0.00	0.00 ± 0.00
α-Terpineol	0.00 ± 0.00	0.00 ± 0.00	0.00 ± 0.00	0.00 ± 0.00	0.00 ± 0.00	0.00 ± 0.00	0.35 ± 0.03	0.00 ± 0.00	0.00 ± 0.00
Others MTs	4.18 ± 0.45	0.00 ± 0.00	2.81 ± 0.19	1.35 ± 0.74	1.02 ± 0.35	1.19 ± 0.29	4.02 ± 1.09	9.85 ± 0.85	5.39 ± 1.51
Guaiadiene	26.50 ± 1.53	14.76 ± 0.45	0.00 ± 0.00	8.77 ± 0.29	0.57 ± 0.07	0.46 ± 0.01	4.63 ± 0.17	0.66 ± 0.04	0.00 ± 0.00
Germacrene D	14.79 ± 1.27	7.66 ± 0.28	1.79 ± 0.29	1.92 ± 0.32	3.67 ± 0.38	3.51 ± 0.3	8.07 ± 0.31	4.7 ± 0.87	2.08 ± 0.16
10-epi-γ-eudesmol	0.00 ± 0.00	0.00 ± 0.00	0.00 ± 0.00	0.00 ± 0.00	6.62 ± 0.07	6.79 ± 0.06	0.00 ± 0.00	0.00 ± 0.00	0.00 ± 0.00
α-Eudesmol	0.00 ± 0.00	0.00 ± 0.00	0.00 ± 0.00	0.03 ± 0.03	0.00 ± 0.00	0.44 ± 0.09	0.00 ± 0.00	2.16 ± 0.19	0.00 ± 0.00
Others STs	23.21 ± 0.29	11.82 ± 0.57	1.97 ± 0.15	6.57 ± 0.94	6.2 ± 0.04	8.2 ± 0.14	29.11 ± 0.55	34.2 ± 0.91	7.26 ± 0.82
Unknowns	2.35 ± 0.30	0.76 ± 0.24	0.00 ± 0.00	1.27 ± 0.45	0.19 ± 0.19	0.28 ± 0.08	0.00 ± 0.00	0.00 ± 0.00	0.00 ± 0.00
**Amount (mg/g)**	**1.93 ± 0.28**	**0.45 ± 0.03**	**1.14 ± 0.14**	**2.77 ± 1.53**	**1.73 ± 0.46**	**2.28 ± 0.38**	**6.24 ± 0.56**	**1.19 ± 0.15**	**0.50 ± 0.13**

*P. cap, P. capitatum; P. grav, P. graveolens; P. quer, P. quercifolium; P. tom, P. tomentosum. Commercial cultivars in brackets; pelargonium rosat cultivars: ‘Bourbon,’ ‘Grasse’ and ‘Egypt’; ‘Clorinda’ is a commercial hybrid from unknown lineage. The two main compounds of each species are shaded. Pinenes, α- and β-pinenes; phellandrenes, α- and β-phellandrenes; ocimenes, α-, β- and allo-ocimenes; menthone, addition of menthone, isomenthone and menthol; citronellol, addition of citronellol, citronellal and several citronellyl like acetate, formate, butyrate and tigliate; geraniol, addition of geraniol, citral, and several geranyl like acetate, formate, butyrate and tigliate; MT, monoterpenes; guaiadiene, 6,9-guaiadiene and 1(5),1,1-guaiadiene; ST, sesquiterpenes. Average percentages and total amounts as well as their corresponding standard deviations were calculated from results obtained from three independent GC-MS analyses.*

**FIGURE 6:**
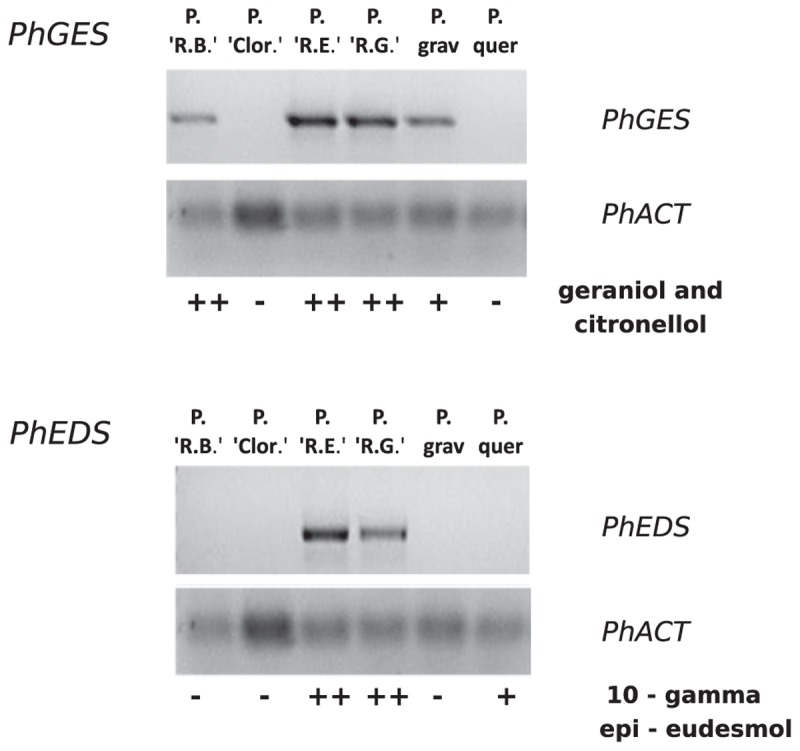
Semi-quantitative RT-PCR analysis of *PhGES* and *PhEDS* in several pelargonium species. *PhACT* is used as a reference gene. Abundance of terpenes related to the expression of each gene is depicted in the lower lane after Table 1.

Expression of both *PhGES* and *PhEDS* was assessed by semi-quantitative RT-PCR using *PhACTIN* as reference (Figure 6). As expected, *PhGES* was expressed in all pelargonium rosat and in *P. graveolens* that produces citronellol and geraniol as main terpenes, *PhEDS* was only expressed in rosat ‘Grasse’ and ‘Egypt’ cultivars that contain 10-γ-epi-eudesmol, Overall, these results indicated a good correlation between *PhGES*, and *PhEDS* gene expression and accumulation of the products synthesised by the respective enzymes in several *Pelargonium* species.

**FIGURE 7:**
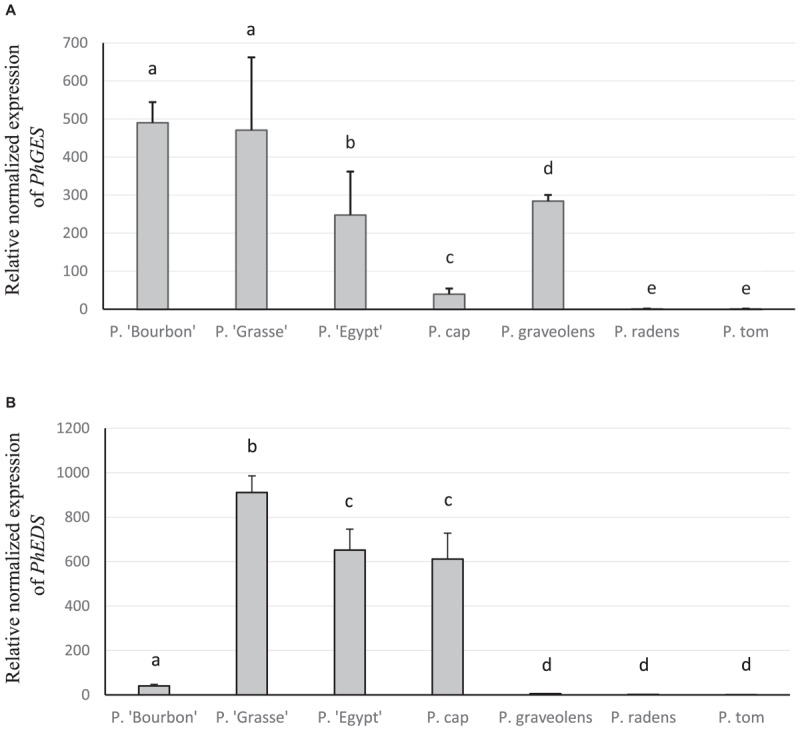
Transcriptional activity of *PhGES* **(A)** and *PhEDS* **(B)** in several pelargonium species. TPS transcript levels were normalised to b*-actin* and *tubulin* then to the expression level of *P. tomentosum*. Error bars indicate standard deviation with *n* = 4. Statistical analyses were performed with the software REST. Same letter indicates no statistical differences with a *p*-value < 0.05.

Expressions of *PhGES* and *PhEDS* genes were more precisely assessed by qPCR. As shown in Figure 7, the results essentially recapitulated the semi-quantitative RT-PCR results. In addition, *PhGES* was only slightly expressed in the species *P. capitatum* (previously not analysed by semi-quantitative RT-PCR). Expression of *PhEDS* in *P. capitatum* was unexpected but similar results were already reported in *Zingiber zerumbet* (Yu et al., 2008). These results confirmed a strong correlation between *PhGES* and *PhEDS* gene expression and accumulation of the products synthesised by the respective enzymes.

### TPS Family in *Pelargonium* Botanical Species and Phylogenetic Relationships of PhGES, PhEDS, PhCINS, and PhMYRS

RNA-Seq data are publically available for 12 botanical species of *Pelargonium* and for *P.* × *hybridum* cv. rosat ‘Bourbon’ (Narnoliya et al., 2017). Hence, this opportunity was used to explore the TPS family in the *Pelargonium* genus, as well as the phylogenetic placement of the four newly discovered enzymes. To these aims, transcriptomes were assembled from the 13 Illumina read sets and annotated. 271 TPS sequences belonging to subfamilies -a, -b, -g, and -e/f (Chen et al., 2011) were retrieved using TPS HMM profile from the Illumina transcriptomes and from the 454 *P.* × *hybridum* cv. rosat ‘Grasse’ transcriptome, thus excluding homologs and some diTPS enzymes belonging to the TPS-c subfamily (see the section “Materials and Methods” for details). The number of TPS sequences by species varied from three enzymes in *P. cotyledonis* to 36 enzymes in *P.* × *hybridum* rosat ‘Grasse’ (Table 2). Because TPS homologs were searched in transcriptome data, the number of TPS retrieved by species is likely underestimated as compared to the total number of TPS encoded by their respective genomes. No clear links could be drawn between the number of transcriptional units per transcriptome and the number of TPS sequences identified (Table 2) showing that the number of TPS per species does not correlate with the number of transcripts inferred during transcriptome assembly. However, analysis of volatile content from available plant specimens in Lyon botanical garden (Supplementary Table 2) showed that the three non-scented species devoid of any detected terpene (*P. cotyledonis*, *P. transvaalense* and *P. australe*) had a low number of TPS sequences in their transcriptome (Table 2). Although the analysed set of plants was limited, this result could mean that non-scented species express or have a lower number of TPS sequences encoded in their genome. It should be noted that the apparent discrepancy in the number of TPSs found between rosat ‘Bourbon’ and rosat ‘Grasse’ cultivars is likely due to the different sequencing and assembly methods used.

**Table 2:** Number of assembled transcripts, number of TPS sequences and number of detected compounds amongst a common set of terpenes between species.

Species	Number of transcriptional units	Number of TPS	Number of terpenes
*P. cotyledonis*	46,138	3	0
*P. transvaalense*	25,683	6	0
*P. myrrhifolium*	22,004	7	N.A.
*P. australe*	32,368	7	0
*P. tetragonum*	24,598	10	11
*P.* × *hybridum* cv. rosat ‘Bourbon’	28,133	16	47^∗∗^
*P. citronellum*	34,842	16	15
*P.* × *hortorum*	47,577	16	17
*P. echinatum*	30,565	23	9
*P. nanum*	35,752	23	N.A.
*P. incrassatum*	27,605	25	N.A.
*P. dichondrifolium*	39,388	25	N.A.
*P. fulgidum*	34,484	26	10
*P. extipulatum*	48,614	32	12
*P.* × *hybridum* cv. rosat ‘Grasse’	225,783^∗^	36	33^∗∗^

*^∗^The 6 to 10 order of magnitude difference in sequence number between the *P.* × hybridum cv. rosat ‘Grasse’ and the other transcriptomes is due to the sequencing technology and assembly procedure. ^∗∗^Both P. × hybridum cv. rosat ‘Bourbon’ and P. × hybridum cv. rosat ‘Grasse’ volatiles were analysed independently of the botanical species.*

**FIGURE 8:**
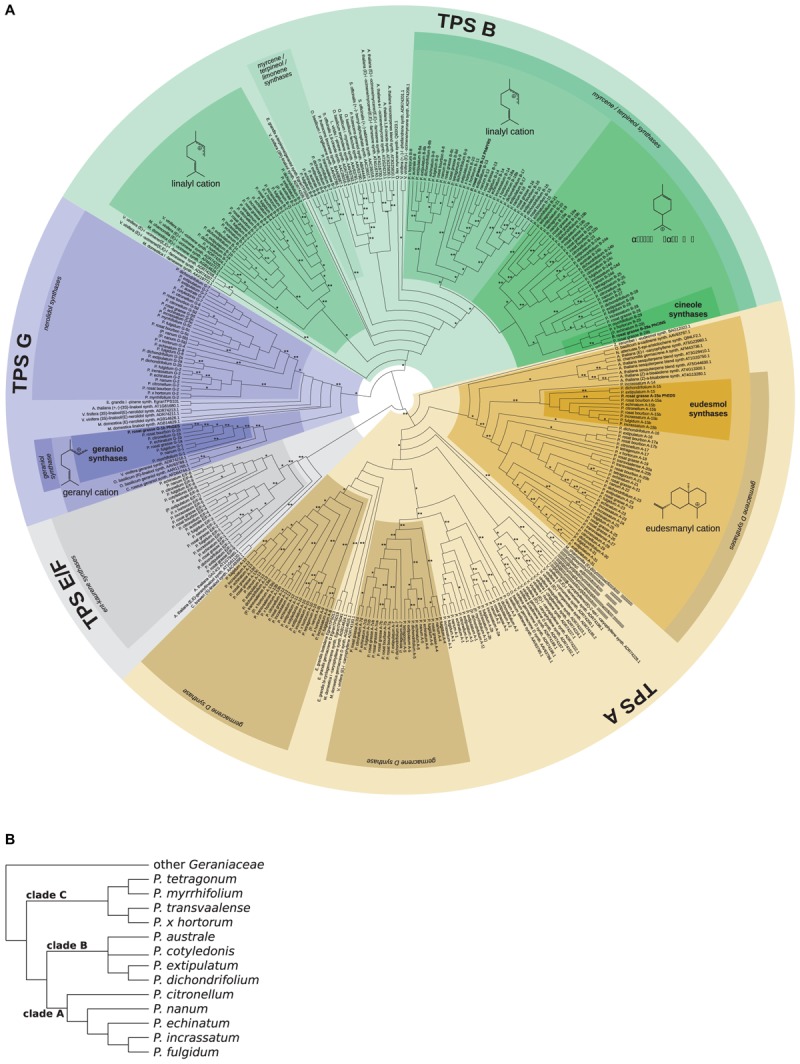
Phylogeny of *Pelargonium* TPS and *Pelargonium* species. Maximum likelihood phylogeny of *Pelargonium* and of functionally characterised TPS from other species **(A)**. Classification of TPS is indicated by concentric circles, from the outside to the inside: TPS subfamilies, annotation from BLAST results, putative intermediary carbocation, and putative function from orthology relationships with functionally characterised enzymes. Carbocations were tentatively deduced from known reaction pathways and functionally characterised enzymes in the clade. Orthology relationships between TPS sequences are indicated in the name of the sequence using the subfamily letter followed by a number. Orthology was reconstructed from the known *Pelargonium* species phylogeny **(B)**, redrawn after (Weng et al., 2012). Some flexibility was allowed within each *Pelargonium* clade and sequences not belonging to the right clade are indicated in brackets. Sequences from pelargonium rosat were considered to cluster with *P. citronellum* sequences. Characterised TPS in this study are indicated in bold. Bootstrap support: ^∗^ ≥70, ^∗∗^ ≥90.

A maximum likelihood (ML) phylogeny (Figure 8A) was inferred including the 271 TPS sequences identified across the set of *Pelargonium* species, the four newly discovered enzymes PhGES, PhCINS, PhMYRS, and PhEDS, as well as *Arabidopsis* sequences and functionally characterised enzymes from diverse species. Two cloned sequences from *P.* × *hybridum* cv. rosat ‘Bourbon’ homologous to PhGES and PhEDS were included in the phylogeny. As expected, PhCINS and PhMYRS clustered with the TPS-b subfamily that contains most of the mTPSs; PhEDS clustered with the TPS-a subfamily made of angiosperms sTPSs; PhGES clustered with the TPS-g subfamily as many other geraniol synthases (Figure 8A). Species phylogeny (Figure 8B) was reconciled with the TPS ML tree in order to infer orthologous groups of TPSs between the 13 *Pelargonium* species. With the exception of PhMYRS for which no orthologs could be inferred, PhGES, PhEDS, and PhCINS possessed orthologous sequences in other *Pelargonium* species, with the orthologous group comprising PhGES (G-1) being strongly supported by bootstrap values (Figure 8A). PhEDS and PhGES possessed direct orthologs in *P.* × *hybridum* rosat ‘Bourbon,’ and PhCINS seemed to be closely related to a paralogous enzyme that consequently could be a second cineole synthase expressed in *P.* × *hybridum* cv. rosat ‘Grasse.’ However, no clear correlation could be drawn between presence or absence of an enzyme in a given species, and terpene content analysis (Supplementary Table 4).

Terpene specificity was tentatively assigned to the *Pelargonium* TPSs, both using blast information and connection of *Pelargonium* TPS clades to functionally characterised TPSs in other species. In almost all cases, functionally characterised enzymes with different product affinity clustered together by species (see *V. vinifera* sequences in the TPS-a subfamily for example, Figure 8A) and branched at the base of clades containing *Pelargonium* TPS sequences, consequently providing no indication as for the putative functions of the *Pelargonium* enzymes. As such, terpene specificity of TPSs has evolved independently in *Pelargonium* genus compared to other lineages. A remarkable exception was represented by the geraniol synthase clade where GESs from different species clustered at the base of the clade containing PhGES orthologous sequences, both forming an independent clade in the TPS-g subfamily (Figure 8A). Thus, GES function seems to be conserved throughout angiosperm evolution, although TPSs functioning as GES evolved also out of the TPS-g subfamily (Yang et al., 2005). The GES group contained also an enzyme acting as a linalool synthase.

## Discussion

### TPS Gene Expression Explain Terpene Diversity in *P. × hybridum* Cultivars and Their Parents

*P. capitatum* in one hand and *P. graveolens* or *P. radens* in the other hand are the putative parents of *P.* × *hybridum* cultivars. *P. radens* and *P graveolen*s have been described as mint-scented species with isomenthone as major compound (Van der Walt and Demarne, 1988), but geraniol and citronellol were reported in EO of some accessions of *P. graveolens* (Lis-Balchin, 2003). Such differences in EO composition could be explained either by wrong botanical identification or by the presence of different chemotypes in the species. This last hypothesis is supported by the fact that in *P. capitatum*, at least 8 chemotypes have been described (Viljoen et al., 1995). In any case, the high number of species in *Pelargonium* genus points out the importance of careful botanical identification of accessions. Several cultivars of pelargonium rosat have been obtained in different places of production. All these cultivars are characterised by a high amount of geraniol, citronellol and other derivative compounds, with different balances depending on the cultivars. In contrast, pelargonium rosat cultivars ‘Grasse’ and ‘Egypt’ can be chemotypically differentiated from ‘Bourbon’ cultivar by their high content in 10-γ-epi-eudesmol.

Expression of *PhGES* and *PhEDS* was studied by semi quantitative RT-PCR and qPCR. A good correlation was found between *PhGES* expression and the accumulation of geraniol and downstream products in the studied *Pelargonium* cultivars. Rosat ‘Bourbon’ and ‘Grasse’ cultivars, the two highest geraniol and citronellol producers, were also those that expressed the highest level of *PhGES*. In contrast, *P. radens* and *P. tomentosum* that did not produce geraniol or citronellol exhibited a low expression of *PhGES*. Transcriptional regulation of TPS genes has already been shown (Nagegowda, 2010), although other modes of regulation cannot be excluded. qPCR results demonstrated that PhGES was implicated in the biosynthesis of geraniol in pelargoniums *in vivo*. *PhEDS* expression was very high in the two cultivars rosat ‘Egypt’ and ‘Grasse,’ both producing a high level of 10-γ-epi-eudesmol, whereas no expression was found in accessions devoid of this terpene, to the exception of *P. capitatum*. In this species, a high level of *PhEDS* transcripts was found but no 10-γ-epi-eudesmol could be detected. A similar unexpected result was previously reported by Yu et al. (2008) who found a high expression of β-eudesmol synthase and no accumulation of the corresponding product. One explanation could be the further conversion of 10-γ-epi-eudesmol to secondary non-volatile derivatives or a lack of FPP substrate. This result remains unclear and needs further investigations.

### PhGES, a Key Enzyme in Controlling the Scent of Pelargonium Rosat Cultivars

Geraniol, citronellol and derivative compounds are the main terpenes involved in the fragrance of *P.* × *hybridum* rosat cultivars, all deriving from the enzymatic activity of PhGES. PhGES belongs to the TPS-g subfamily. Enzymes from this clade lack the RR(x_8_)W conserved motif, which facilitates isomerisation of the geranyl cation in the linalyl cation, a step necessary to form cyclic terpenes (Williams et al., 1998). Thus, TPS-g enzymes can only produce acyclic terpenes. Although the first characterised enzyme from this clade was involved in monoterpene synthesis, it is known that this clade gathers mono, sesqui and diterpenes synthases catalysing acyclic terpene biosynthesis. PhGES is well conserved in the different rosat cultivars, as well as in their putative parents, although some do not produce geraniol. Interestingly, PhGES sequence possessed three supplementary amino acids (TAL) in rosat ‘Bourbon’ and ‘Egypt’ cultivars, as compared to rosat ‘Grasse’ cultivar. This three amino acids were also found in *P. graveolens* species. Taken together, this indicate that pelargonium rosat ‘Grasse’ cultivar could be descending from a different crossing as compared to the two other rosats. GES sequence in *P.* cv. ‘Toussaint,’ a hybrid cultivated for its high content in citronellol, was more different compared to rosat cultivars and *P. graveolens*, clearly indicating a different origin.

### PhEDS, a New Sesquiterpene Synthase

**FIGURE 9:**
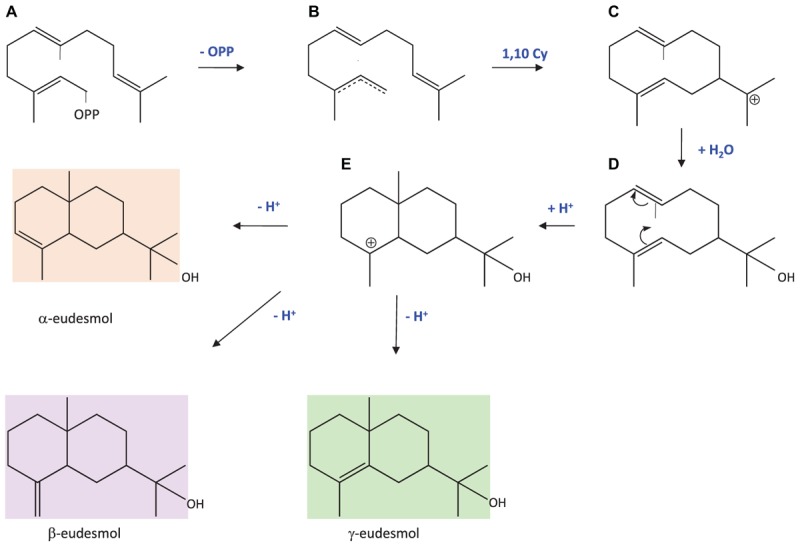
Biosynthetic pathway leading to eudesmols modified from Rabe et al. (2016). **(A)** Farnesyl diphosphate; **(B)** farnesyl cation; **(C)** (*E,E*) germacradienyl, **(D)** hedycaryol; **(E)** eudesmol cation.

Eudesmols are sesquiterpenes that are biosynthesised from a farnesyl cation that undergoes a 1,10 cyclisation leading to the germacradienyl cation (Figure 9). A nucleophile attack of the cation by water leads first to the stabilisation of the molecule by addition of a hydroxyl group. Then, mesmerisation between the two double bonds allows an internal 2,7 closure with a protonation producing another carbocation intermediate. Finally, a deprotonation stabilises the molecule, restoring a double bond at a position depending on the position of the lost proton, consequently producing different eudesmol isomers. In this paper, we provide the first cloning and functional characterisation of a 10-epi-γ-eudesmol synthase (PhEDS). To our knowledge, one β-eudesmol synthase from ginger catalysing the formation of both α- and β-eudesmol has previously been reported (Yu et al., 2008). β-eudesmol was shown to confer resistance to plants against ant attack (Marinho et al., 2005) and to possess antifungal activity (Guleria et al., 2012). Although eudesmol production was enhanced by abiotic stresses in pelargonium rosat cultivars (Blerot, 2016), it is difficult to assess yet the specific role of 10-epi-γ-eudesmol in these plants without further investigations.

### TPS Evolution in the *Pelargonium* Genus

Analysis of the *P.* × *hybridum* rosat ‘Grasse,’ *P.* × *hybridum* rosat ‘Bourbon,’ and the 12 botanical *Pelargonium* transcriptomes unveiled about 270 TPS sequences from the *Pelargonium* genus. The terpene specificity of most of these enzymes cannot be assigned on the sole base of sequence homology nor phylogenetic relationships. This is due to the rapid evolution by duplication and mutation acquisition of TPS sequences (Chen et al., 2011; Jullien et al., 2014) leading to convergent functional evolution in this family at low phylogenetic levels. Incidentally, functional plasticity of TPS enzymes has been demonstrated experimentally by target mutations of the active site (Yoshikuni et al., 2006; Kampranis et al., 2007). As a consequence, TPS sequences with different enzymatic capabilities from close species are more related to each other than homologous sequences with the same enzymatic specificities from more distantly related species, as is exemplified here by the *Pelargonium* centric TPS phylogeny. It should be noted, however, that this plasticity crosses TPS subfamilies borders only marginally: for example, sTPSs are predominantly found within the TPS-a subfamily whereas mTPSs are for the most part found within the TPS-b subfamily. Contrasting with this observation, closely related paralogs from the same subfamily can use both GPP and FPP as a substrate in *in vitro* assays but produce only monoterpenes or sesquiterpenes *in planta* because of their differential subcellular localisation, either in the chloroplast or in the cytosol where only GPP or FPP is available, respectively (Nagegowda et al., 2008; Huang et al., 2010). Moreover, synthesis of some terpene like linalool can be ensured by enzymes from diverse origins, see for example the linalool synthases sequences placed in TPS-g, -b and -e/f subfamilies in Figure 8A and Magnard et al. (2018). Geraniol synthesis, however, seems to be mainly catalysed by a group of enzymes clustering in a clade localised within the TPS-g subfamily, although enzymes from other subfamilies have evolved as GES (for example the *P. frutescens* GES belongs to the TPS-b subfamily, see Figure 8A). This observation asks why GES enzymes from the TPS-g subfamily keep their product specificity across larger phylogenetic distances as compared to others TPSs. A possible explanation could be that TPS-g enzymes have limited evolutionary routes because of their inability to form cyclic terpenes, as described above.

Orthologous sequences to three of the four newly discovered TPS enzymes in *P.* × *hybridum* rosat ‘Grasse’ could be identified by reconciling species phylogeny with the *Pelargonium* TPS phylogeny. It is tempting to assign the same terpene specificity to the *P.* × *hybridum* rosat ‘Grasse’ PhGES, PhEUS, and PhCINS respective orthologs, although one should stay cautious in such a functional assignment given the fast evolution of TPS sequences, as discussed above. Presence of theses enzymes and of their orthologs does not always correlate with terpene composition observed in the different *Pelargonium* species. This apparent discrepancy can be easily explained by several factors: expression level of the TPS gene, posttranscriptional regulation, utilisation of the enzyme product by subsequent reactions, absence of the substrate at the time of the chemical analysis, chemical analysis sensitivity, etc.

## Conclusion

In this study, we identified and functionally characterised for the first time a GES and an EDS in pelargonium, two key enzymes controlling scent and EO composition of this odorant and economically important plant. We characterised two other mTPSs and placed the four enzymes in an evolutionary perspective of the TPS family within the *Pelargonium* genus, thus laying the foundation of a better understanding of how pelargonium odour is produced. Functional characterisation of more TPS in *Pelargonium* and finer chemical analysis will provide a better understanding of TPS evolution within the genus and, more generally, will allow gaining insights in how odorant *Pelargonium* species evolved and could be used as new sources of genetic material for perfumery industry. To this aim, large scale multiomic studies of several odorant cultivars and species combining transcriptomics and terpene metabolomics will bring to light entire metabolic pathways involved in the synthesis of compounds making the richness of fragrance observed in *Pelargonium*.

## Author Contributions

BB and LM brought equivalent contribution to the experimental work and deserve therefore to be both ranked as first author. CP and AB gave a large contribution in molecular cloning of pelargonium TPSs. DS-M and SL performed the bioinformatics analysis. LS, FG, and NB were involved in the project as gardeners to maintain the pelargonium collection in greenhouse. SB, J-CC, and FJ supervised the experiments. FJ and DS-M wrote the manuscript. BB, LM, DS-M, FJ, SB, and J-CC contributed to the discussion and interpretation of the results and read and approved the final manuscript.

## Conflict of Interest Statement

The authors declare that the research was conducted in the absence of any commercial or financial relationships that could be construed as a potential conflict of interest.

## Footnotes

1 http://www.bioinformatics.babraham.ac.uk/projects/fastqc/

2 http://www.arabidopsis.org/

3 https://www.ncbi.nlm.nih.gov/sra

4 https://github.com/TransDecoder/TransDecoder

## References

[B1] Andersen C. L. Jensen J. L. Ørntoft T. F. (2004). Normalization of real-time quantitative reverse transcription-PCR data: a model-based variance estimation approach to identify genes suited for normalization, applied to bladder and colon cancer data sets. *Cancer Res.* 64 5245–5250. 10.1158/0008-5472.CAN-04-0496 15289330

[B2] Bakker F. T. Culham A. de Marais A. B. Gibby M. (2005). “Nested radiation in cape *Pelargonium*,” in Plant Species-Level Systematics: New Perspectives on Pattern & Process, *Koeltz Scientific Books*, eds Bakker F. T. Chatrou L. W. (Wageningen: Wageningen University), 75–100.

[B3] Bakker F. T. Culham A. Hettiarachi P. Touloumenidou T. Gibby M. (2004). Phylogeny of *Pelargonium* (Geraniaceae) based on DNA sequences from three genomes. *Taxon* 53 17–31. 10.2307/4135485 10811797

[B4] Batoko H. Zheng H.-Q. Hawes C. Moore I. (2000). A Rab1 GTPase is required for transport between the endoplasmic reticulum and golgi apparatus and for normal golgi movement in plants. *Plant Cell* 12 2201–2217. 10.1105/tpc.12.11.2201 11090219 PMC150168

[B5] Bertea C. M. Schalk M. Karp F. Maffei M. Croteau R. (2001). Demonstration that menthofuran synthase of mint (Mentha) is a cytochrome P450 monooxygenase: cloning, functional expression, and characterization of the responsible gene. *Arch. Biochem. Biophys.* 390 279–286. 10.1006/abbi.2001.2378 11396930

[B6] Blerot B. (2016). *Biosynthèse des Composés Odorants Chez Différents Pelargonium Utilisés Pour la Production d’huile Essentielle.* PhD thesis, University of Saint-Etienne, Saint-Etienne.

[B7] Blerot B. Baudino S. Prunier C. Demarne F. Toulemonde B. Caissard J.-C. (2015). Botany, agronomy and biotechnology of *Pelargonium* used for essential oil production. *Phytochem. Rev.* 5 935–960.

[B8] Bohlmann J. Meyer-Gauen G. Croteau R. (1998). Plant terpenoid synthases: molecular biology and phylogenetic analysis. *Proc. Natl. Acad. Sci. U.S.A.* 95 4126–4133. 10.1073/pnas.95.8.4126 9539701 PMC22453

[B9] Bolger A. M. Lohse M. Usadel B. (2014). Trimmomatic: a flexible trimmer for Illumina sequence data. *Bioinformatics* 30 2114–2120. 10.1093/bioinformatics/btu170 24695404 PMC4103590

[B10] Bonnot C. Proust H. Pinson B. Colbalchini F. P. L. Lesly-Veillard A. Breuninger H. (2017). Functional PTB phosphate transporters are present in streptophyte algae and early diverging land plants. *New Phytol.* 214 1158–1171. 10.1111/nph.14431 28134432

[B11] Boukhatem M. N. Kameli A. Saidi F. (2013). Essential oil of algerian rose-scented geranium (*Pelargonium graveolens*): chemical composition and antimicrobial activity against food spoilage pathogens. *Food Control* 34 208–213. 10.1016/j.foodcont.2013.03.045

[B12] Boukhris M. Nasri-Ayachi M. B. Mezghani I. Bouaziz M. Boukhris M. Sayadi S. (2013). Trichomes morphology, structure and essential oils of *Pelargonium graveolens* L’Hér. (Geraniaceae). *Ind. Crops Prod.* 50 604–610. 10.1016/j.indcrop.2013.08.029

[B13] Bradford M. M. (1976). A rapid and sensitive method for the quantitation of microgram quantities of protein utilizing the principle of protein-dye binding. *Anal. Biochem.* 72 248–254. 10.1016/0003-2697(76)90527-3 942051

[B14] Busso D. Delagoutte-Busso B. Moras D. (2005). Construction of a set gateway-based destination vectors for high-throughput cloning and expression screening in *Escherichia coli*. *Anal. Biochem.* 343 313–321. 10.1016/j.ab.2005.05.015 15993367

[B15] Chang S. Puryear J. Cairney J. (1993). A simple and efficient method for isolating RNA from pine trees. *Plant Mol. Biol. Rep.* 11 113–116. 10.1007/BF02670468 11725489

[B16] Chen F. Tholl D. Bohlmann J. Pichersky E. (2011). The family of terpene synthases in plants: a mid-size family of genes for specialized metabolism that is highly diversified throughout the kingdom. *Plant J.* 66 212–229. 10.1111/j.1365-313X.2011.04520.x 21443633

[B17] Chen X. Köllner T. G. Jia Q. Norris A. Santhanam B. Rabe P. (2016). Terpene synthase genes in eukaryotes beyond plants and fungi: occurrence in social amoebae. *Proc. Natl. Acad. Sci.* *U.S.A*. 113 12132–12137. 10.1073/pnas.1610379113 27790999 PMC5087028

[B18] Cock J. M. Swarup R. Dumas C. (1997). Natural antisense transcripts of the S locus receptor kinase gene and related sequences in *Brassica oleracea*. *Mol. Genet. MGG* 255 514–524. 10.1007/s004380050524 9294036

[B19] Crocoll C. Asbach J. Novak J. Gershenzon J. Degenhardt J. (2010). Terpene synthases of oregano (*Origanum vulgare* L.) and their roles in the pathway and regulation of terpene biosynthesis. *Plant Mol. Biol.* 73 587–603. 10.1007/s11103-010-9636-1 20419468

[B20] Curtis M. D. Grossniklaus U. (2003). A gateway cloning vector set for high-throughput functional analysis of genes in planta. *Plant Physiol.* 133 462–469. 10.1104/pp.103.027979 14555774 PMC523872

[B21] Darriba D. Taboada G. L. Doallo R. Posada D. (2011). ProtTest 3: fast selection of best-fit models of protein evolution. *Bioinformatics* 27 1164–1165. 10.1093/bioinformatics/btr088 21335321 PMC5215816

[B22] Davis E. M. Ringer K. L. McConkey M. E. Croteau R. (2005). Monoterpene metabolism. Cloning, expression, and characterization of menthone reductases from peppermint. *Plant Physiol.* 137 873–881. 10.1104/pp.104.053306 15728344 PMC1065388

[B23] Degenhardt J. Köllner T. G. Gershenzon J. (2009). Monoterpene and sesquiterpene synthases and the origin of terpene skeletal diversity in plants. *Evol. Metab. Divers.* 70 1621–1637. 10.1016/j.phytochem.2009.07.030 19793600

[B24] Demarne F.-E. (2003). “Rose-scented geranium a *Pelargonium* grown for the perfume industry,” in *Geranium and Pelargonium* ed. Lis-Balchin M. (London: South Bank University), 193–211.

[B25] Demarne F. E. Van der Walt J. J. A. (1992). Composition of the essential oil of *Pelargonium vitifolium* (L.) L’Herit.(Geraniaceae). *J. Essent. Oil Res.* 4 345–348. 10.1080/10412905.1992.9698083

[B26] Dugé de Bernonville T. Clastre M. Besseau S. Oudin A. Burlat V. Glévarec G. (2015). Phytochemical genomics of the Madagascar periwinkle: unravelling the last twists of the alkaloid engine. *Phytochemistry* 113 9–23. 10.1016/j.phytochem.2014.07.023 25146650

[B27] Eddy S. R. (2011). Accelerated profile HMM Searches. *PLoS Comput. Biol.* 7:e1002195. 10.1371/journal.pcbi.1002195 22039361 PMC3197634

[B28] Emanuelsson O. Brunak S. Heijne H. von Nielsen G. (2007). Locating proteins in the cell using TargetP, SignalP and related tools. *Nat. Protoc.* 2 953–971. 10.1038/nprot.2007.131 17446895

[B29] Gang D. R. Wang J. Dudareva N. Nam K. H. Simon J. E. Lewinsohn E. (2001). An investigation of the storage and biosynthesis of phenylpropenes in sweet basil. *Plant Physiol.* 125 539–555. 10.1104/pp.125.2.539 11161012 PMC64856

[B30] Gauvin A. Lecomte H. Smadja J. (2004). Comparative investigations of the essential oils of two scented geranium (*Pelargonium* spp.) cultivars grown on Reunion Island. *Flavour Fragr. J.* 19 455–460. 10.1002/ffj.1354

[B31] Guleria S. Tiku A. K. Gupta S. Singh G. Koul A. Razdan V. K. (2012). Chemical composition, antioxidant activity and inhibitory effects of essential oil of *Eucalyptus teretecornis* grown in north-western Himalaya against *Alternaria alternata*. *J. Plant Biochem. Biotechnol.* 21 44–50. 10.1007/s13562-011-0073-2

[B32] Hassan M. MaaroF N. D. Ali Z. M. Noor N. M. Othman R. Mori N. (2012). Monoterpene alcohol metabolism: identification, purification, and characterization of two geraniol dehydrogenase isoenzymes from *Polygonum minus* Leaves. *Biosci. Biotechnol. Biochem.* 76 1463–1470. 10.1271/bbb.120137 22878188

[B33] Hayashi K. Kawaide H. Notomi M. Sakigi Y. Matsuo A. Kawai H. (2006). Identification and functional analysis of bifunctional ent-kaurene synthase from the moss *Physcomitrella* patens. *FEBS Lett.* 580 6175–6181. 10.1016/j.febslet.2006.10.018 17064690

[B34] Huang M. Abel C. Sohrabi R. Petri J. Haupt I. Cosimano J. (2010). Variation of herbivore-induced volatile terpenes among *Arabidopsis* ecotypes depends on allelic differences and subcellular targeting of two terpene synthases, TPS02 and TPS03. *Plant Physiol.* 153 1293–1310. 10.1104/pp.110.154864 20463089 PMC2899926

[B35] Huang X. Madan A. (1999). CAP3: a DNA sequence assembly program. *Genome Res.* 9 868–877. 10.1101/gr.9.9.868 10508846 PMC310812

[B36] Iijima Y. Koeduka T. Suzuki H. Kubota K. (2014). Biosynthesis of geranial, a potent aroma compound in ginger rhizome (*Zingiber officinale*): molecular cloning and characterization of geraniol dehydrogenase. *Plant Biotechnol.* 31 525–534. 10.5511/plantbiotechnology.14.1020a

[B37] Iijima Y. Wang G. Fridman E. Pichersky E. (2006). Analysis of the enzymatic formation of citral in the glands of sweet basil. *Arch Biochem Biophys.* 448 141–149. 10.1016/j.abb.2005.07.026 16150417

[B38] Jadaun J. S. Sangwan N. S. Narnoliya L. K. Singh N. Bansal S. Mishra B. (2017). Over-expression of DXS gene enhances terpenoidal secondary metabolite accumulation in rose-scented geranium and *Withania somnifera*: active involvement of plastid isoprenogenic pathway in their biosynthesis. *Physiol. Plant.* 159 381–400. 10.1111/ppl.12507 27580641

[B39] Jones C. S. Bakker F. T. Schlichting C. D. Nicotra A. B. (2009). Leaf shape evolution in the south African genus *Pelargonium* l’Her. (*Geraniaceae)*. *Evolution* 63 479–497. 10.1111/j.1558-5646.2008.00552.x 19154370

[B40] Juliani H. R. Koroch A. Simon J. E. Hitimana N. Daka A. Ranarivelo L. (2006). Quality of geranium oils (*Pelargonium* species): case studies in Southern and Eastern Africa. *J. Essent. Oil Res.* 18 116–121.

[B41] Jullien F. Moja S. Bony A. Legrand S. Petit C. Benabdelkader T. (2014). Isolation and functional characterization of a τ-cadinol synthase, a new sesquiterpene synthase from Lavandula angustifolia. *Plant Mol. Biol.* 84 227–241. 10.1007/s11103-013-0131-3 24078339

[B42] Kampranis S. C. Ioannidis D. Purvis A. Mahrez W. Ninga E. Katerelos N. A. (2007). Rational conversion of substrate and product specificity in a salvia monoterpene synthase: structural insights into the evolution of terpene synthase function. *Plant Cell* 19 1994–2005. 10.1105/tpc.106.047779 17557809 PMC1955729

[B43] Lalli J. Y. Viljoen A. M. Başer K. H. C. Demirci B. Özek T. (2006). The essential oil composition and chemotaxonomical appraisal of south african *Pelargoniums* (Geraniaceae). *J. Essent. Oil Res.* 18 89–105.

[B44] Lane A. Boecklemann A. Woronuk G. N. Sarker L. Mahmoud S. S. (2010). A genomics resource for investigating regulation of essential oil production in *Lavandula angustifolia*. *Planta* 231 835–845. 10.1007/s00425-009-1090-4 20043174

[B45] Lange B. M. Wildung M. R. Stauber E. J. Sanchez C. Pouchnik D. Croteau R. (2000). Probing essential oil biosynthesis and secretion by functional evaluation of expressed sequence tags from mint glandular trichomes. *Proc. Natl. Acad. Sci.* *U.S.A.* 97 2934–2939. 10.1073/pnas.97.6.2934 10717007 PMC16033

[B46] Lis-Balchin M. (2003). “Essential oils from different *Pelargonium* species and cultivars: their chemical composition (using GC, GC/MS) and appearance of trichomes (under EM),” in *Geranium and Pelargonium*, ed. Lis-Balchin M. (London: South Bank University), 147–165.

[B47] Livak K. J. Schmittgen T. D. (2001). Analysis of relative gene expression data using real-time quantitative PCR and the 2^−ΔΔCT^ method. *Methods* 25 402–408. 10.1006/meth.2001.1262 11846609

[B48] Magnard J.-L. Bony A. R. Bettini F. Campanaro A. Blerot B. Baudino S. (2018). Linalool and linalool nerolidol synthases in roses, several genes for little scent. *Plant Physiol. Biochem.* 127 74–87. 10.1016/j.plaphy.2018.03.009 29550664

[B49] Magnard J.-L. Roccia A. Caissard J.-C. Vergne P. Sun P. Hecquet R. (2015). Biosynthesis of monoterpene scent compounds in roses. *Science* 349 81–83. 10.1126/science.aab0696 26138978

[B50] Marinho C. G. S. Della Lucia T. M. C. Guedes R. N. C. Ribeiro M. M. R. Lima E. R. (2005). β-eudesmol-induced aggression in the leaf-cutting ant *Atta sexdens* rubropilosa. *Entomol. Exp. Appl.* 117 89–93. 10.1111/j.1570-7458.2005.00338.x

[B51] Martin D. M. Aubourg S. Schouwey M. B. Daviet L. Schalk M. Toub O. (2010). Functional annotation, genome organization and phylogeny of the grapevine (*Vitis vinifera*) terpene synthase gene family based on genome assembly, FLcDNA cloning, and enzyme assays. *BMC Plant Biol.* 10:226. 10.1186/1471-2229-10-226 20964856 PMC3017849

[B52] Mendoza-Poudereux I. Kutzner E. Huber C. Segura J. Eisenreich W. Arrillaga I. (2015). Metabolic cross-talk between pathways of terpenoid backbone biosynthesis in spike lavender. *Plant Physiol. Biochem.* 95 113–120. 10.1016/j.plaphy.2015.07.029 26254184

[B53] Muñoz-Bertomeu J. Arrillaga I. Ros R. Segura J. (2006). Up-regulation of 1-deoxy-D-xylulose-5-phosphate synthase enhances production of essential oils in transgenic spike lavender. *Plant Physiol.* 142 890–900. 10.1104/pp.106.086355 16980564 PMC1630752

[B54] Nagegowda D. A. (2010). Plant volatile terpenoid metabolism: biosynthetic genes, transcriptional regulation and subcellular compartmentation. *FEBS Lett.* 584 2965–2973. 10.1016/j.febslet.2010.05.045 20553718

[B55] Nagegowda D. A. Gutensohn M. Wilkerson C. G. Dudareva N. (2008). Two nearly identical terpene synthases catalyze the formation of nerolidol and linalool in snapdragon flowers. *Plant J.* 55 224–239. 10.1111/j.1365-313X.2008.03496.x 18363779

[B56] Narnoliya L. K. Kaushal G. Singh S. P. Sangwan R. S. (2017). De novo transcriptome analysis of rose-scented geranium provides insights into the metabolic specificity of terpene and tartaric acid biosynthesis. *BMC Genomics* 18:74. 10.1186/s12864-016-3437-0 28086783 PMC5234130

[B57] Opitz S. Nes W. D. Gershenzon J. (2014). Both methylerythritol phosphate and mevalonate pathways contribute to biosynthesis of each of the major isoprenoid classes in young cotton seedlings. *Phytochemistry* 98 110–119. 10.1016/j.phytochem.2013.11.010 24359633

[B58] Pfaffl M. W. Horgan G. W. Dempfle L. (2002). Relative expression software tool (REST∖copyright) for group-wise comparison and statistical analysis of relative expression results in real-time PCR. *Nucleic Acids Res.* 30:e36 10.1093/nar/30.9.e36 PMC113859 11972351

[B59] Pfaffl M. W. Tichopad A. Prgomet C. Neuvians T. P. (2004). Determination of stable housekeeping genes, differentially regulated target genes and sample integrity: bestKeeper–Excel-based tool using pair-wise correlations. *Biotechnol. Lett.* 26 509–515. 10.1023/B:BILE.0000019559.84305.47 15127793

[B60] Price M. N. Dehal P. S. Arkin A. P. (2010). FastTree 2–approximately maximum-likelihood trees for large alignments. *PLoS One* 5:e9490. 10.1371/journal.pone.0009490 20224823 PMC2835736

[B61] Rabe P. Schmitz T. Dickschat S. (2016). Mechanistic investigations on six bacterial terpene cyclases, Beilstein *J. Org. Chem.* 12 1839–1850. 27829890 10.3762/bjoc.12.173 PMC5082573

[B62] Rao B. R. Kaul P. N. Syamasundar K. V. Ramesh S. (2002). Water soluble fractions of rose-scented geranium (*Pelargonium* species) essential oil. *Bioresour. Technol.* 84 243–246. 10.1016/S0960-8524(02)00057-3 12118700

[B63] Ringer K. L. Davis E. M. Croteau R. (2005). Monoterpene metabolism. Cloning, expression, and characterization of (-)-isopiperitenol/(-)-carveol dehydrogenase of peppermint and spearmint. *Plant Physiol.* 137 863–872. 10.1104/pp.104.053298 15734920 PMC1065387

[B64] Ringer K. L. McConkey M. E. Davis E. M. Rushing G. W. Croteau R. (2003). Monoterpene double-bond reductases of the (-)-menthol biosynthetic pathway: isolation and characterization of cDNAs encoding (-)-isopiperitenone reductase and (+)-pulegone reductase of peppermint. *Arch. Biochem. Biophys.* 418 80–92. 10.1016/S0003-9861(03)00390-4 13679086

[B65] Roeschenbleck J. Albers F. Müller K. Weinl S. Kudla J. (2014). Phylogenetics, character evolution and a subgeneric revision of the genus *Pelargonium* (Geraniaceae). *Phytotaxa* 159 31–76. 10.11646/phytotaxa.159.2.1

[B66] Sangwan R. Singh U. (2015). *A Process for the Production of Natural and Scented Tartaric Acid from Geranium (Pelargonium graveolens) Biomass:Geranium Biomass Hydro-Distillation Residual Water as a Novel Biomass.* Patent Application No. 1487/DEL/2015.

[B67] Saraswathi J. Venkatesh K. Baburao N. Hilal M. H. Rani A. R. (2011). Phytopharmacological importance of *Pelargonium* species. *J. Med. Plants Res.* 5 2587–2598. 29378636

[B68] Sato-Masumoto N. Ito M. (2014). Two types of alcohol dehydrogenase from *Perilla* can form citral and perillaldehyde. *Phytochemistry* 104 12–20. 10.1016/j.phytochem.2014.04.019 24864017

[B69] Shalit M. Guterman I. Volpin H. Bar E. Tamari T. Menda N. (2003). Volatile ester formation in roses. Identification of an acetyl-coenzyme A. Geraniol/Citronellol acetyltransferase in developing rose petals. *Plant Physiol.* 131 1868. 10.1104/pp.102.018572 12692346 PMC166943

[B70] Sievers F. Wilm A. Dineen D. Gibson T. J. Karplus K. Li W. (2011). Fast, scalable generation of high-quality protein multiple sequence alignments using Clustal Omega. *Mol. Syst. Biol.* 7:539. 10.1038/msb.2011.75 21988835 PMC3261699

[B71] Simpson J. T. Durbin R. (2011). Efficient de novo assembly of large genomes using compressed data structures. *Genome Res.* 22 549–556. 10.1101/gr.126953.111 22156294 PMC3290790

[B72] Small I. Peeters N. Legeai F. Lurin C. (2004). Predotar: a tool for rapidly screening proteomes for N-terminal targeting sequences. *Proteomics* 4 1581–1590. 10.1002/pmic.200300776 15174128

[B73] Stamatakis A. (2014). RAxML version 8: a tool for phylogenetic analysis and post-analysis of large phylogenies. *Bioinformatics* 30 1312–1313. 10.1093/bioinformatics/btu033 24451623 PMC3998144

[B74] Tholl D. Chen F. Petri J. Gershenzon J. Pichersky E. (2005). Two sesquiterpene synthases are responsible for the complex mixture of sesquiterpenes emitted from *Arabidopsis* flowers. *Plant J.* 42 757–771. 10.1111/j.1365-313X.2005.02417.x 15918888

[B75] Van der Walt J. J. A. Demarne F. E. (1988). Pelargonium graveolens and *P*. *radens*: a comparison of their morphology and essential oils. *South Afr. J. Bot.* 54 617–622. 10.1016/S0254-6299(16)31263-7

[B76] Vandesompele J. De Preter K. Pattyn F. Poppe B. Van Roy N. De Paepe A. (2002). Accurate normalization of real-time quantitative RT-PCR data by geometric averaging of multiple internal control genes. *Genome Biol.* 3:0034-31. 12184808 10.1186/gb-2002-3-7-research0034 PMC126239

[B77] Viljoen A. M. Van der Walt J. J. A. Swart J. P. J. Demame F.-E. (1995). A study of the variation in the essential oil of *Pelargonium capitatum* (L.) L’Herit.(Geraniaceae). Part II. The Chemotypes of *P capitatum*. *J. Essent. Oil Res.* 7 605–611. 10.1080/10412905.1995.9700514 22906882

[B78] Voinnet O. Rivas S. Mestre P. Baulcombe D. (2003). An enhanced transient expression system in plants based on suppression of gene silencing by the p19 protein of tomato bushy stunt virus. *Plant J.* 33 949–956. 10.1046/j.1365-313X.2003.01676.x 12609035

[B79] Wang Q. Reddy V. A. Panicker D. Mao H.-Z. Kumar N. Rajan C. (2016). Metabolic engineering of terpene biosynthesis in plants using a trichome-specific transcription factor MsYABBY5 from spearmint (*Mentha spicata*). *Plant Biotechnol. J.* 14 1619–1632. 10.1111/pbi.12525 26842602 PMC5067620

[B80] Wawrzyn G. T. Bloch S. E. Schmidt-Dannert C. (2012). “Discovery and characterization of terpenoid biosynthetic pathways of fungi,” in *Methods in Enzymology Natural Product Biosynthesis by Microorganisms and Plants, Part A.*, ed. Hopwood D. A. (Cambridge, MA: Academic Press), 83–105. 10.1016/B978-0-12-394290-6.00005-7 22999171

[B81] Weng M.-L. Ruhlman T. Gibby M. Jensen R. K. (2012). Phylogeny, rate variation and, genome size evolution of *Pelargonium* (Geraniaceae). *Mol. Phylogenet. Evol.* 64 654–670. 10.1016/j.ympev.2012.05.026 22677167

[B82] Williams D. C. McGarvey D. J. Katahira E. J. Croteau R. (1998). Truncation of limonene synthase preprotein provides a fully active ‘Pseudomature’ form of this monoterpene cyclase and reveals the function of the amino-terminal arginine pair. *Biochemistry (Mosc.)* 37 12213–12220. 10.1021/bi980854k 9724535

[B83] Xu H. Bohman B. Wong D. C. Rodriguez-Delgado C. Scaffidi A. Flematti G. R. (2017). Complex sexual deception in an orchid is achieved by co-opting two independent biosynthetic pathways for pollinator attraction. *Curr. Biol.* 27 1867–1877. 10.1016/j.cub.2017.05.065 28625782

[B84] Yamada Y. Kuzuyama T. Komatsu M. Shin-ya K. Omura S. Cane D. E. (2015). Terpene synthases are widely distributed in bacteria. *Proc. Natl. Acad. Sci. U.S.A.* 112 857–862. 10.1073/pnas.1422108112 25535391 PMC4311827

[B85] Yang T. Li J. Wang H.-X. Zeng Y. (2005). A geraniol-synthase gene from *Cinnamomum tenuipilum*. *Phytochemistry* 66 285–293. 10.1016/j.phytochem.2004.12.004 15680985

[B86] Yoshikuni Y. Ferrin T. E. Keasling J. D. (2006). Designed divergent evolution of enzyme function. *Nature* 440 1078–1082. 10.1038/nature04607 16495946

[B87] Yu F. Harada H. Yamasaki K. Okamoto S. Hirase S. Tanaka Y. (2008). Isolation and functional characterization of a β-eudesmol synthase, a new sesquiterpene synthase from Zingiber zerumbet Smith. *FEBS Lett.* 582 565–572. 10.1016/j.febslet.2008.01.020 18242187

