# Supplementary material for: Functional Analysis of Four Terpene Synthases in Rose-Scented Pelargonium Cultivars (Pelargonium × hybridum) and Evolution of Scent in the Pelargonium Genus
Source: Front Plant Sci. 2018 Nov 2;9:1435. doi: 10.3389/fpls.2018.01435 (PMC6240891; doi:10.3389/fpls.2018.01435)
Supplement: Supplementary file 1 [file Presentation_1.pptx]

## Slide 1
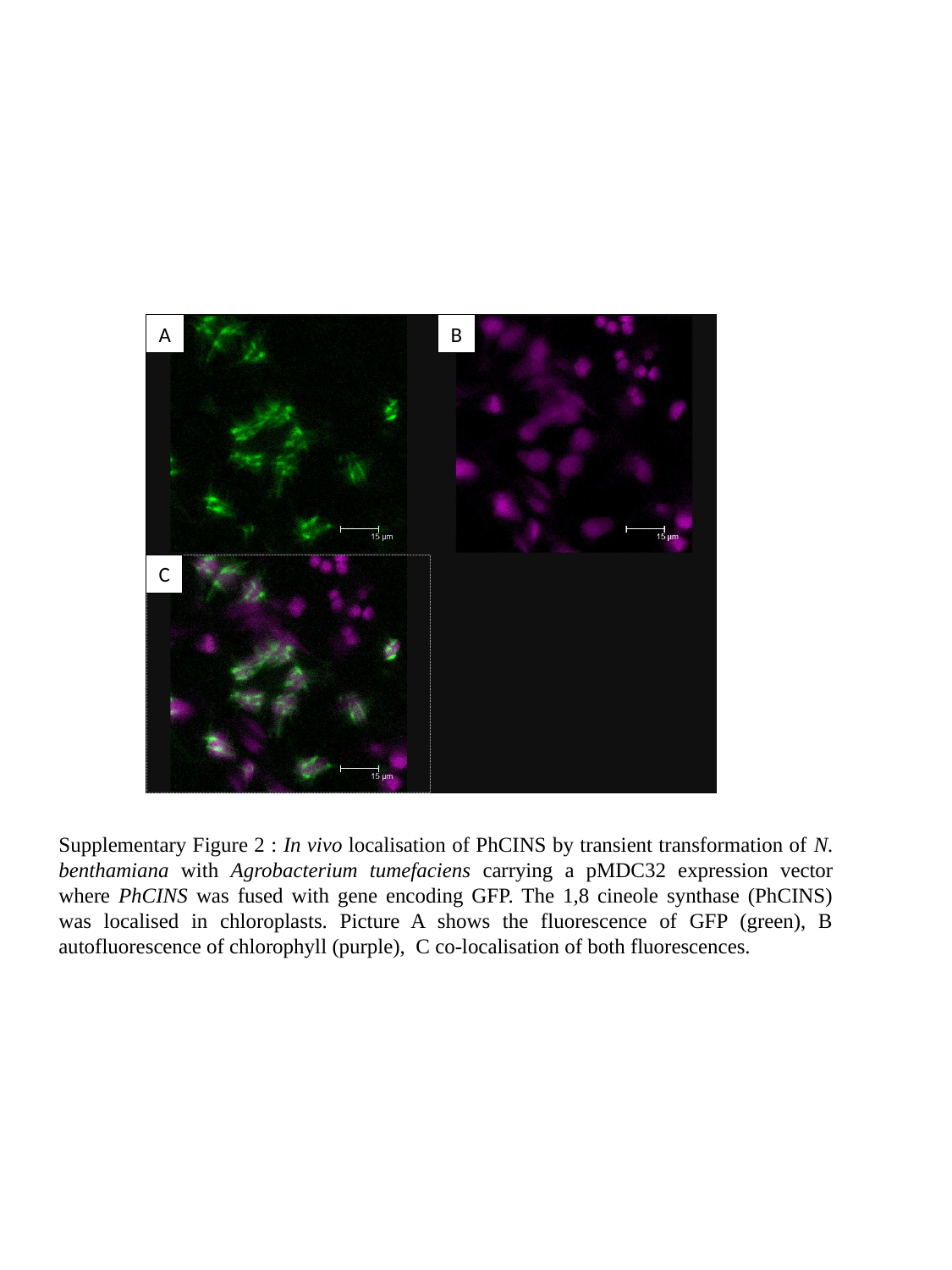

A
B
C
Supplementary Figure 2 : In vivo localisation of PhCINS by transient transformation of N. benthamiana with Agrobacterium tumefaciens carrying a pMDC32 expression vector where PhCINS was fused with gene encoding GFP. The 1,8 cineole synthase (PhCINS) was localised in chloroplasts. Picture A shows the fluorescence of GFP (green), B autofluorescence of chlorophyll (purple), C co-localisation of both fluorescences.
