# Supplementary material for: Functional Analysis of Four Terpene Synthases in Rose-Scented Pelargonium Cultivars (Pelargonium × hybridum) and Evolution of Scent in the Pelargonium Genus
Source: Front Plant Sci. 2018 Nov 2;9:1435. doi: 10.3389/fpls.2018.01435 (PMC6240891; doi:10.3389/fpls.2018.01435)
Supplement: Supplementary file 4 [file Table_2.DOCX]

Supplementary Table 2: Accession, terpene product, species and associated publication of functionally characterised TPS used in the phylogenetic tree.

| **accession** | **database** | **terpene product(s)** | **species** | **publication** |
| --- | --- | --- | --- | --- |
| AT4G16730.1 | TAIR | E-b-ocimene\|myrcene\|ee-a-farnesene | *Arabidopsis thaliana* | (Huang et al., 2010) |
| AT4G16740.1 | TAIR | E-b-ocimene\|myrcene\|ee-a-farnesene |  |  |
| AT1G61120.1 | TAIR | GES_ee-geranyllinalool |  | (Herde et al., 2008) |
| AT2G24210.1 | TAIR | e-b-ocimene\|myrcene |  | (Bohlmann et al., 2000) |
| AT4G13280.1 | TAIR | z-g-bisabolene |  | (Ro et al., 2006) |
| AT4G13300.1 | TAIR | z-g-bisabolene |  |  |
| AT1G61680.1 | TAIR | +-3s-linalool |  | (Chen et al., 2003) |
| AT5G23960.1 | TAIR | e-b-caryophyllene |  | (Tholl et al., 2005) |
| AT3G25830.1 | TAIR | 1-8-cineole |  | (Chen et al., 2004) |
| AT5G44630.1 | TAIR | sesqui-blend |  | (Tholl et al., 2005) |
| AT1G33750.1 | TAIR | sesqui-blend |  | (Wang et al., 2016) |
| AT3G25810.1 | TAIR | mono-blend |  | (Chen et al., 2003) |
| AT3G29410.1 | TAIR | sesqui-blend |  | (Wang et al., 2016) |
| AT1G79460.1 | TAIR | GA2_KS |  | (Yamaguchi et al., 1998) |
| AFM43734.1 | NCBI | E-b-caryophyllene | *Matricaria chamomilla* | (Irmisch et al., 2012) |
| AFM43735.1 | NCBI | a-isocomene |  |  |
| AFM43736.1 | NCBI | germacrene-A |  |  |
| AFM43737.1 | NCBI | A-b-ocimene |  |  |
| AFM43738.1 | NCBI | germacrene-D |  |  |
| AAC26018.1 | NCBI | +-sabinene | *Salvia officinalis* | (Wise et al., 1998) |
| AAC26017.1 | NCBI | +-bornyl-diphosphate |  |  |
| AAC26016.1 | NCBI | 1-8-cineole |  |  |
| AAC49395.1 | NCBI | S-linalool | *Clarkia breweri* | (Dudareva et al., 1996) |
| Q93X23.1 | NCBI | myrcene | *Quercus ilex* | (Fischbach et al., 2001) |
| BAA08367.1 | NCBI | limonene | *Perilla frutescens* | (Yuba et al., 1996) |
| ABI54448.1 | NCBI | geraniol |  | (Ito and Honda, 2007) |
| AFD64744.1 | NCBI | geraniol | *Catharanthus roseus* | (Simkin et al., 2013) |
| AAX19772.1 | NCBI | alpha-farnesene | *Malus domestica* | (Green et al., 2007) |
| AGB14624.1 | NCBI | beta-caryophyllene |  | (Nieuwenhuizen et al., 2013) |
| AGB14625.1 | NCBI | germacrene-D |  |  |
| AGB14626.1 | NCBI | E-nerolidol |  |  |
| AGB14627.1 | NCBI | pinene\|camphene |  |  |
| AGB14628.1 | NCBI | E-beta-ocimene |  |  |
| AGB14629.1 | NCBI | linalool |  |  |
| AGN72805.1 | NCBI | a-pinene | *Lavandula viridis* | (Benabdelkader et al., 2015) |
| BAG12022.1 | NCBI | b-eudesmol | *Zingiber zerumbet* | (Yu et al., 2008) |
| AAV63785.1 | NCBI | selinene | *Ocimum basilicum* | (Iijima et al., 2004a) |
| AAV63786.1 | NCBI | germacrene-D |  |  |
| AAV63790.1 | NCBI | fenchol |  |  |
| AAV63791.1 | NCBI | b-myrcene |  |  |
| AAV63792.1 | NCBI | terpinolene |  |  |
| AAV63788.1 | NCBI | a-zingiberene |  |  |
| AAR11765.1 | NCBI | geraniol |  | (Iijima et al., 2004b) |
| AAV63789.1 | NCBI | R-linalool |  | (Iijima et al., 2004a) |
| AAV63787.1 | NCBI | g-cadinene |  |  |
| Q84LF2.1 | NCBI | 5-epi-aristolochene | *Nicotiana attenuata* | (Bohlmann et al., 2002) |
| EgranTPS041 / Eucgr.K03518.1 | eucgenie | bicyclogermacrene | *Eucalyptus grandis* | (Külheim et al., 2015) |
| EgranTPS013 / Eucgr.D01105.1 | eucgenie | isoledene |  |  |
| EgranTPS059 / ? | eucgenie | g-terpinene_geraniol |  |  |
| EgranTPS019 / Eucgr.E00419.1 | eucgenie | germacrene-D |  |  |
| EgranTPS101 / Eucgr.E03562.1 | eucgenie | b-pinene |  |  |
| ADR74193.1 | NCBI | E-beta-caryophyllene | *Vitis vinifera* | (Martin et al., 2010) |
| ADR74192.1 | NCBI | E-beta-caryophyllene |  |  |
| ADR74222.1 | NCBI | E-beta-caryophyllene |  |  |
| ADR74196.1 | NCBI | germacrene-D |  |  |
| ADR74195.2 | NCBI | E-alpha-bergamotene |  |  |
| ADR74199.1 | NCBI | gamma-cadinene |  |  |
| ADR74197.1 | NCBI | germacrene-D |  |  |
| ADR74198.1 | NCBI | E_E-alpha-farnesene |  |  |
| ADR74200.1 | NCBI | beta-curcumene |  |  |
| ADR74223.1 | NCBI | sesquithujene |  |  |
| ADR74224.1 | NCBI | alpha-zingiberene |  |  |
| ADR74225.1 | NCBI | selina-411-diene/intermedeol |  |  |
| ADR74226.1 | NCBI | cubebol/delta-cadinene |  |  |
| ADR74227.1 | NCBI | alpha-humulene |  |  |
| ADR74228.1 | NCBI | E-beta-caryophyllene/2-epi-E-beta-caryophyllene |  |  |
| ADR74201.1 | NCBI | +-alpha-phellandrene |  |  |
| ADR74204.1 | NCBI | E-beta-ocimene |  |  |
| ADR74205.1 | NCBI | E-beta-ocimene |  |  |
| ADR74206.1 | NCBI | E-beta-ocimene/myrcene |  |  |
| ADR74207.1 | NCBI | E-beta-ocimene/E_E-alpha-farnesene |  |  |
| ADR74208.1 | NCBI | E-beta-ocimene/E_E-alpha-farnesene |  |  |
| ADR74209.1 | NCBI | 3R-linalool |  |  |
| ADR74211.1 | NCBI | 3S-linalool/E-nerolidol |  |  |
| ADR74213.1 | NCBI | 3S-linalool/E-nerolidol |  |  |
| ADR74218.1 | NCBI | geraniol |  |  |

Benabdelkader, T., Guitton, Y., Pasquier, B., Magnard, J.L., Jullien, F., Kameli, A., and Legendre, L. (2015). Functional characterization of terpene synthases and chemotypic variation in three lavender species of section Stoechas. Physiol. Plant. *153*, 43–57.

Bohlmann, J., Martin, D., Oldham, N.J., and Gershenzon, J. (2000). Terpenoid Secondary Metabolism in Arabidopsis thaliana: cDNA Cloning, Characterization, and Functional Expression of a Myrcene/(E)-β-Ocimene Synthase. Arch. Biochem. Biophys. *375*, 261–269.

Bohlmann, J., Stauber, E.J., Krock, B., Oldham, N.J., Gershenzon, J., and Baldwin, I.T. (2002). Gene expression of 5-epi-aristolochene synthase and formation of capsidiol in roots of Nicotiana attenuata and N. sylvestris. Phytochemistry *60*, 109–116.

Chen, F., Tholl, D., D’Auria, J.C., Farooq, A., Pichersky, E., and Gershenzon, J. (2003). Biosynthesis and Emission of Terpenoid Volatiles from Arabidopsis Flowers. Plant Cell *15*, 481–494.

Chen, F., Ro, D.-K., Petri, J., Gershenzon, J., Bohlmann, J., Pichersky, E., and Tholl, D. (2004). Characterization of a Root-Specific Arabidopsis Terpene Synthase Responsible for the Formation of the Volatile Monoterpene 1,8-Cineole. Plant Physiol. *135*, 1956–1966.

Dudareva, N., Cseke, L., Blanc, V.M., and Pichersky, E. (1996). Evolution of floral scent in Clarkia: novel patterns of S-linalool synthase gene expression in the C. breweri flower. Plant Cell *8*, 1137–1148.

Fischbach, R.J., Zimmer, W., and Schnitzler, J.-P. (2001). Isolation and functional analysis of a cDNA encoding a myrcene synthase from holm oak (Quercus ilex L.). Eur. J. Biochem. *268*, 5633–5638.

Green, S., Friel, E.N., Matich, A., Beuning, L.L., Cooney, J.M., Rowan, D.D., and MacRae, E. (2007). Unusual features of a recombinant apple α-farnesene synthase. Phytochemistry *68*, 176–188.

Herde, M., Gärtner, K., Köllner, T.G., Fode, B., Boland, W., Gershenzon, J., Gatz, C., and Tholl, D. (2008). Identification and Regulation of TPS04/GES, an Arabidopsis Geranyllinalool Synthase Catalyzing the First Step in the Formation of the Insect-Induced Volatile C16-Homoterpene TMTT. Plant Cell *20*, 1152–1168.

Huang, M., Abel, C., Sohrabi, R., Petri, J., Haupt, I., Cosimano, J., Gershenzon, J., and Tholl, D. (2010). Variation of Herbivore-Induced Volatile Terpenes among Arabidopsis Ecotypes Depends on Allelic Differences and Subcellular Targeting of Two Terpene Synthases, TPS02 and TPS03. Plant Physiol. *153*, 1293–1310.

Iijima, Y., Davidovich-Rikanati, R., Fridman, E., Gang, D.R., Bar, E., Lewinsohn, E., and Pichersky, E. (2004a). The Biochemical and Molecular Basis for the Divergent Patterns in the Biosynthesis of Terpenes and Phenylpropenes in the Peltate Glands of Three Cultivars of Basil. Plant Physiol. *136*, 3724–3736.

Iijima, Y., Gang, D.R., Fridman, E., Lewinsohn, E., and Pichersky, E. (2004b). Characterization of Geraniol Synthase from the Peltate Glands of Sweet Basil. Plant Physiol. *134*, 370–379.

Irmisch, S., Krause, S.T., Kunert, G., Gershenzon, J., Degenhardt, J., and Köllner, T.G. (2012). The organ-specific expression of terpene synthase genes contributes to the terpene hydrocarbon composition of chamomile essential oils. BMC Plant Biol. *12*, 84.

Ito, M., and Honda, G. (2007). Geraniol synthases from perilla and their taxonomical significance. Phytochemistry *68*, 446–453.

Külheim, C., Padovan, A., Hefer, C., Krause, S.T., Köllner, T.G., Myburg, A.A., Degenhardt, J., and Foley, W.J. (2015). The Eucalyptus terpene synthase gene family. BMC Genomics *16*, 450.

Martin, D.M., Aubourg, S., Schouwey, M.B., Daviet, L., Schalk, M., Toub, O., Lund, S.T., and Bohlmann, J. (2010). Functional Annotation, Genome Organization and Phylogeny of the Grapevine (Vitis vinifera) Terpene Synthase Gene Family Based on Genome Assembly, FLcDNA Cloning, and Enzyme Assays. BMC Plant Biol. *10*, 226.

Nieuwenhuizen, N.J., Green, S.A., Chen, X., Bailleul, E.J.D., Matich, A.J., Wang, M.Y., and Atkinson, R.G. (2013). Functional Genomics Reveals That a Compact Terpene Synthase Gene Family Can Account for Terpene Volatile Production in Apple1[W]. Plant Physiol. *161*, 787–804.

Ro, D.-K., Ehlting, J., Keeling, C.I., Lin, R., Mattheus, N., and Bohlmann, J. (2006). Microarray expression profiling and functional characterization of AtTPS genes: Duplicated Arabidopsis thaliana sesquiterpene synthase genes At4g13280 and At4g13300 encode root-specific and wound-inducible (Z)-γ-bisabolene synthases. Arch. Biochem. Biophys. *448*, 104–116.

Simkin, A.J., Miettinen, K., Claudel, P., Burlat, V., Guirimand, G., Courdavault, V., Papon, N., Meyer, S., Godet, S., St-Pierre, B., et al. (2013). Characterization of the plastidial geraniol synthase from Madagascar periwinkle which initiates the monoterpenoid branch of the alkaloid pathway in internal phloem associated parenchyma. Phytochemistry *85*, 36–43.

Tholl, D., Chen, F., Petri, J., Gershenzon, J., and Pichersky, E. (2005). Two sesquiterpene synthases are responsible for the complex mixture of sesquiterpenes emitted from Arabidopsis flowers. Plant J. *42*, 757–771.

Wang, Q., Jia, M., Huh, J.-H., Muchlinski, A., Peters, R.J., and Tholl, D. (2016). Identification of a Dolabellane Type Diterpene Synthase and other Root-Expressed Diterpene Synthases in Arabidopsis. Front. Plant Sci. *7*.

Wise, M.L., Savage, T.J., Katahira, E., and Croteau, R. (1998). Monoterpene Synthases from Common Sage (Salvia officinalis) cDNA ISOLATION, CHARACTERIZATION, AND FUNCTIONAL EXPRESSION OF (+)-SABINENE SYNTHASE, 1,8-CINEOLE SYNTHASE, AND (+)-BORNYL DIPHOSPHATE SYNTHASE. J. Biol. Chem. *273*, 14891–14899.

Yamaguchi, S., Sun, T., Kawaide, H., and Kamiya, Y. (1998). The GA2 Locus of Arabidopsis thalianaEncodes ent-Kaurene Synthase of Gibberellin Biosynthesis. Plant Physiol. *116*, 1271–1278.

Yu, F., Harada, H., Yamasaki, K., Okamoto, S., Hirase, S., Tanaka, Y., Misawa, N., and Utsumi, R. (2008). Isolation and functional characterization of a β-eudesmol synthase, a new sesquiterpene synthase from Zingiber zerumbet Smith. FEBS Lett. *582*, 565–572.

Yuba, A., Yazaki, K., Tabata, M., Honda, G., and Croteau, R. (1996). cDNA Cloning, Characterization, and Functional Expression of 4S-(−)-Limonene Synthase fromPerilla frutescens. Arch. Biochem. Biophys. *332*, 280–287.
