# Supplementary material for: Functional Analysis of Four Terpene Synthases in Rose-Scented Pelargonium Cultivars (Pelargonium × hybridum) and Evolution of Scent in the Pelargonium Genus
Source: Front Plant Sci. 2018 Nov 2;9:1435. doi: 10.3389/fpls.2018.01435 (PMC6240891; doi:10.3389/fpls.2018.01435)
Supplement: Supplementary file 5 [file Table_3.DOCX]

Supplementary Table 3: Genomic structure of a geraniol synthase (*PhGES)* and a 10-epi-γ-eudesmol synthase (*PhEDS)* obtained from *P. x hybridum* cv.’Grasse’. The length of introns (I-VII) and exons (1-8) is indicated by the number of bases.

| Gene | 1 | I | 2 | II | 3 | III | 4 | IV | 5 | V | 6 | VI | 7 | VII | 8 | Total |
| --- | --- | --- | --- | --- | --- | --- | --- | --- | --- | --- | --- | --- | --- | --- | --- | --- |
| *PhGES* | 165 | 118 | 274 | 440 | 331 | 542 | 219 | 73 | 139 | 238 | 249 | 561 | 291 |  |  | 3640 |
| *PhEDS* | 129 | 183 | 283 | 96 | 376 | 129 | 219 | 162 | 139 | 94 | 249 | 89 | 311 | 75 | 147 | 2681 |
